# Quaternary cellulose adsorbents for environmental applications: preparation, properties, and future prospects

**DOI:** 10.1039/d6ra03001g

**Published:** 2026-08-20

**Authors:** Bartosz Fabiszczak, Roksana Markiewicz

**Affiliations:** a NanoBioMedical Centre, Adam Mickiewicz University Wszechnicy Piastowskiej 3 Poznan 61-614 Poznań Poland roksana.markiewicz@amu.edu.pl; b Faculty of Chemistry, Adam Mickiewicz University Uniwersytetu Poznańskiego 8 Poznan 61-614 Poznań Poland

## Abstract

Cellulose, the most abundant natural polymer, has gained notable attention in recent years for environmental applications owing to its biodegradability, renewability, and ease of modification. Among cellulose derivatives, quaternary cellulose has become a promising adsorbent for removing a wide range of environmental pollutants. Although numerous studies report high adsorption capacities, the practical application of quaternary cellulose is limited by challenges related to material stability and upscaling. This review critically analyses recent advances in the synthesis, characterization, and environmental performance of various quaternary cellulose structures. The capacity of these materials to adsorb anionic compounds, their adsorption mechanisms, and crucial limitations in regeneration efficiency are discussed. Key challenges that need to be addressed before large-scale implementation are identified, including the demand for systematic studies on long-term performance, environmental impact, and economic effectiveness.

## Abstract 2

Cellulose, the most abundant natural polymer, has gained notable attention in recent years for environmental applications owing to its biodegradability, renewability, and ease of modification.

## Graphical abstract

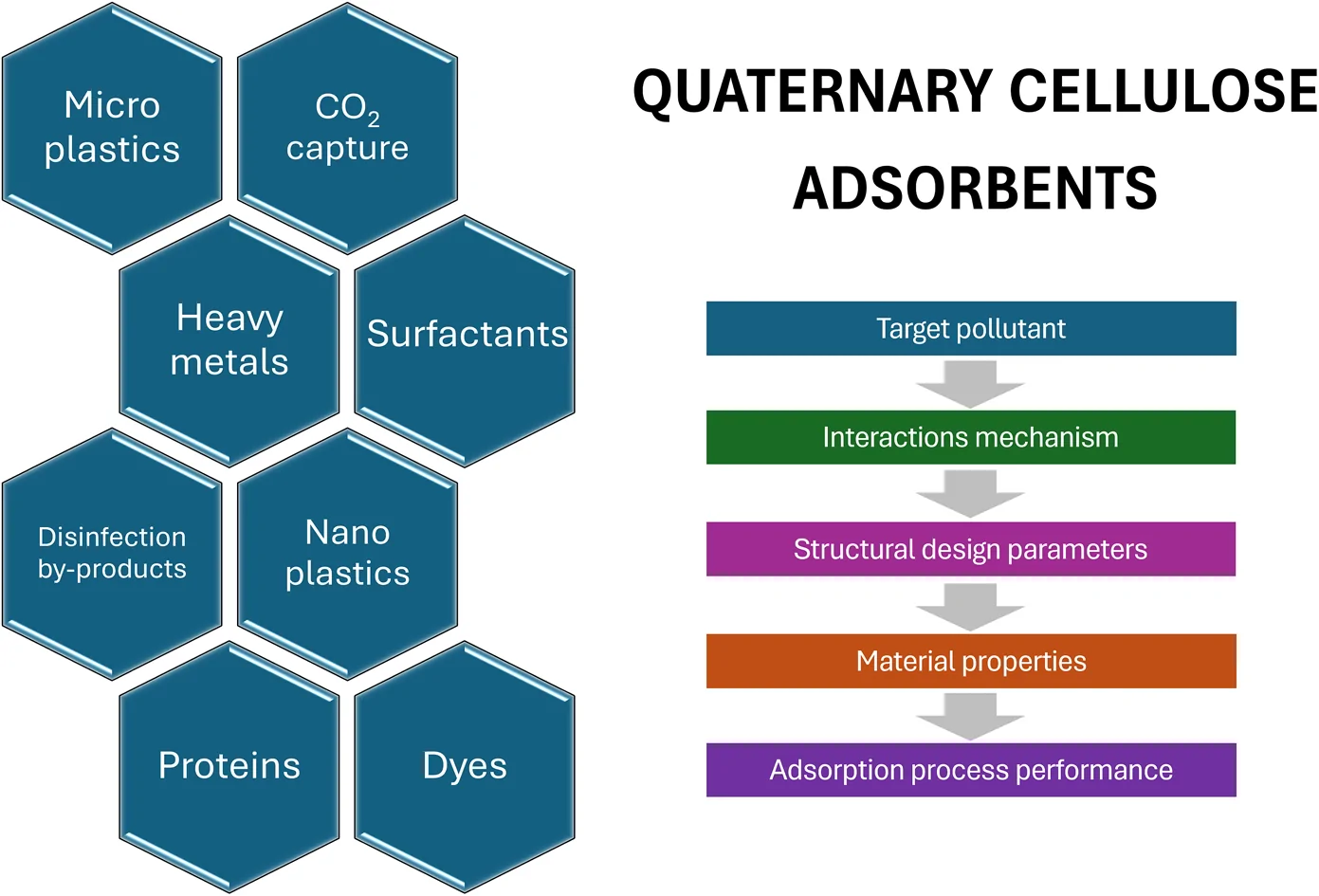

## Introduction

Increasing living standards have a significant impact on the environment overall and on water quality. The most pressing challenges stem from industrial, agricultural, and domestic activities, which together significantly degrade air, water, and soil quality. Severe pollution is caused mainly by large-scale releases from households and post-industrial wastewater. Additionally, habitat degradation, the impacts of climate change, and the overuse of freshwater resources, along with numerous environmental disasters, are emerging and are never environmentally neutral.^1,2^ The purity and safety of water resources are crucial to human and animal health.

The development of sustainable adsorbents for water treatment has attracted increasing attention in response to the growing need for materials that combine high removal efficiency with low environmental burden. Among the variety of water purification methods, including chemical, physical, mechanical, and biological treatments, adsorption technologies are operationally simple, effective, cost-efficient, and applicable to diverse classes of pollutants. Recent years have seen many potential adsorbents emerge; at the same time, the growing emphasis on renewable and biodegradable materials has intensified interest in polysaccharide-based adsorbents, especially those derived from cellulose. Considering the environmental perspective and guided by the principles of green chemistry, materials that effectively remove a variety of contaminants from water sources should be more sustainable and safer.^3–5^

In that manner, natural polysaccharides are inexpensive, biocompatible, biodegradable, and made from natural, renewable resources.^6,7^ However, pristine cellulose has important limitations as an adsorbent. Its surface chemistry is dominated by hydroxyl groups, and its extensive intra- and intermolecular hydrogen-bonding network restricts both solubility and chemical accessibility. As a result, the adsorption performance of unmodified cellulose is often insufficient to efficiently remove many ionic pollutants, particularly under complex aqueous conditions.^8,9^ To overcome these limitations, cellulose is frequently functionalized to introduce new surface groups and tune its interaction with target contaminants. Among the available modification routes, quaternization is particularly important because it introduces permanent cationic groups, thereby enhancing cellulose's affinity for negatively charged species. Depending on the synthetic route, quaternized cellulose can be prepared as aerogels, hydrogels, films, fibers, beads, membranes, or other porous architectures. This combination of structural tunability and cationic surface chemistry has made quaternized cellulose an increasingly studied class of adsorbents for the removal of dyes, surfactants, disinfection by-products, selected metal-containing species, proteins, carbon dioxide, and microplastics.^10^ Quaternized cellulose, depending on the alkyl chain length, can also increase antibacterial activity, which is advantageous in water treatment applications.^11,12^

The growing body of literature on quaternary cellulose highlights its multifaceted potential in environmental applications. Key aspects of quaternary cellulose include its greenness and sustainability, high stability, versatility in the morphology and chemical structure of the quaternary moiety, biocompatibility, and a wide range of applications.^13^ Quaternary cellulose can be obtained in the forms of aerogels, hydrogels, films, fibres, or other particles with micro- or sub-microscale dimensions. Moreover, various quaternization methods enable the combination of cellulose's sustainability with the ability to modify surface chemistry (tailoring alkyl chain length or selecting the appropriate anion), making it tuneable to specific technological and environmental challenges.^14^ All things considered, quaternized cellulose appears to be an extremely useful type of adsorbent.^15,16^

Despite developments in quaternary cellulose, the field remains fragmented. The studies reported in the literature differ not only in cellulose source, quaternization chemistry, and material morphology, but also in the types of pollutants, testing conditions, regeneration strategies, and performance, making comparison difficult. Moreover, in the literature, the trend is to emphasize adsorption capacity under simplified laboratory conditions, whereas issues important for real-world application, including structural stability, regeneration efficiency, long-term durability, performance in complex water systems, scalability, and environmental cost of functionalization, are addressed much less systematically. For this reason, the practical significance of quaternized cellulose cannot be judged from adsorption capacity alone. Although reviews are available on cellulose-based adsorbents and modified nanocellulose systems, a critical review specifically focused on quaternized cellulose adsorbents, covering synthesis, adsorption performance, regeneration, and translational limitations, remains lacking. In this work, we aimed to provide an extensive evaluation of quaternary cellulose structures, with a specific focus on their environmental applications. Rather than treating quaternized cellulose as a uniformly successful class of materials, we examine how synthetic strategy influences structure, how the structure affects the adsorption process, and how it affects regeneration, durability, and practical use. We described various routes for modification, adsorption studies, and potential applications for the removal of anionic compounds, dyes, surfactants, heavy metals, carbon dioxide, proteins, and microplastics. Our goal is to demonstrate the potential of quaternary cellulose materials for environmental remediation, while highlighting the key challenges that must be overcome for real systems and the need for systematic studies to address durability, environmental impact, and financial feasibility.

## Cellulose, its modifications, and applications

The most abundant naturally occurring polysaccharide is cellulose.^17^ It is one of the essential substances found in organisms such as plants, bacteria, tunicates, algae, and fungi.^18^ The overall cellulose concentration in plants is about 33%. As a linear biopolymer, cellulose consists of d-glucose units connected by β-1,4-glycosidic bonds.^19^ Crystalline cellulose comes in four different allomorphs (named I–IV). Because it is a naturally occurring substance with a crystalline structure and a highly ordered content, cellulose I is extremely resistant to degradation. It is composed of parallel chains of cellulose connected by multiple intermolecular and intramolecular hydrogen bonds. This arrangement makes it an ideal material for maintaining the plant's structural integrity and resisting strain. When Cellulose I undergoes chemical reactions, such as mercerization or exposure to strong acids, Cellulose II is formed. It is amorphous and less structured (a lack of hydrogen bonds results in a reduction in the structure's ordering). Due to its high sensitivity to chemical reactions, this type of cellulose is used to produce various cellulose-based products. After amines are added to cellulose I or II, cellulose III is produced, which again is differently structured and possesses much lower crystallinity. After cellulose III is subjected to extremely high temperatures and glycerol treatment, cellulose IV is produced. The following two allomorphs are less common in nature, whereas the first two are prevalent.^20^Fig. 1 shows the synthesis of different types of cellulose from Cellulose I.

**Fig. 1:**
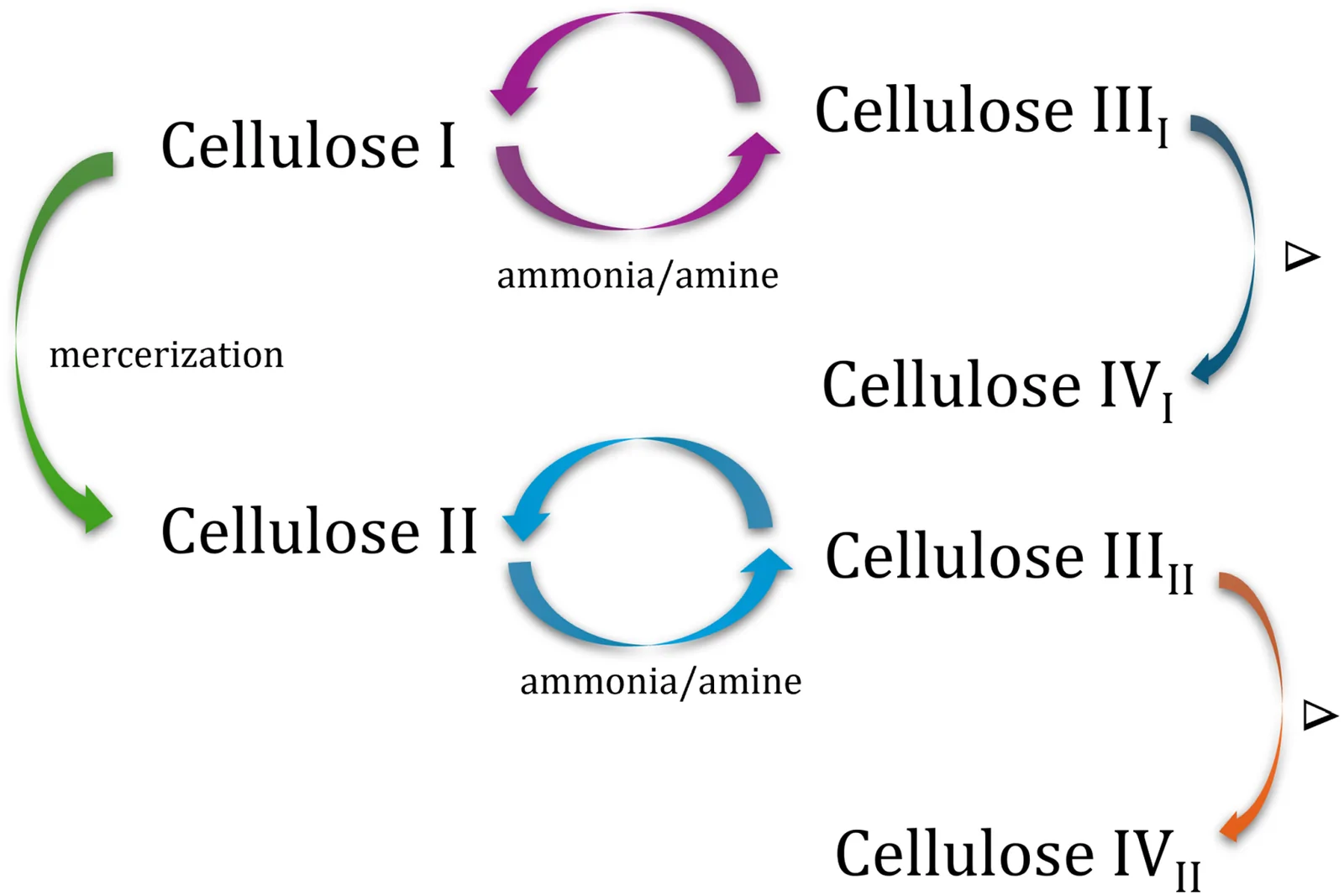
Various allomorphs of cellulose.

As a naturally occurring substance, cellulose is highly biodegradable; it has high mechanical strength, a large specific surface area, and low density, which makes it useful in numerous and diverse applications.^21^ Importantly, one can distinguish between two types of cellulose: bacterial and plant.^22^ Several types of bacteria, including *Acetobacter xylinum*, can synthesize bacterial cellulose. Plant cellulose is extracted from agricultural and other waste materials, such as sugar cane bagasse, soybean husks, pear peels, jute, wheat straw, and many more. Bacterial cellulose is usually characterized by higher purity and homogeneity than plant cellulose, despite having a less organized crystal structure. Bacterial cellulose is utilized in the food and pharmaceutical industries, while plant cellulose is more frequently found in the paper and textile fields.^23^ Plant cellulose derived from agricultural waste is abundant worldwide and easily available. These resources are almost inexhaustible, not to mention their low price. This makes the preparation of diverse nanostructures low-cost; moreover, such waste can be reused in multiple applications, making the process economical and environmentally friendly.^24^ A scheme of the types of nanocellulose is shown in Fig. 2.

**Fig. 2:**
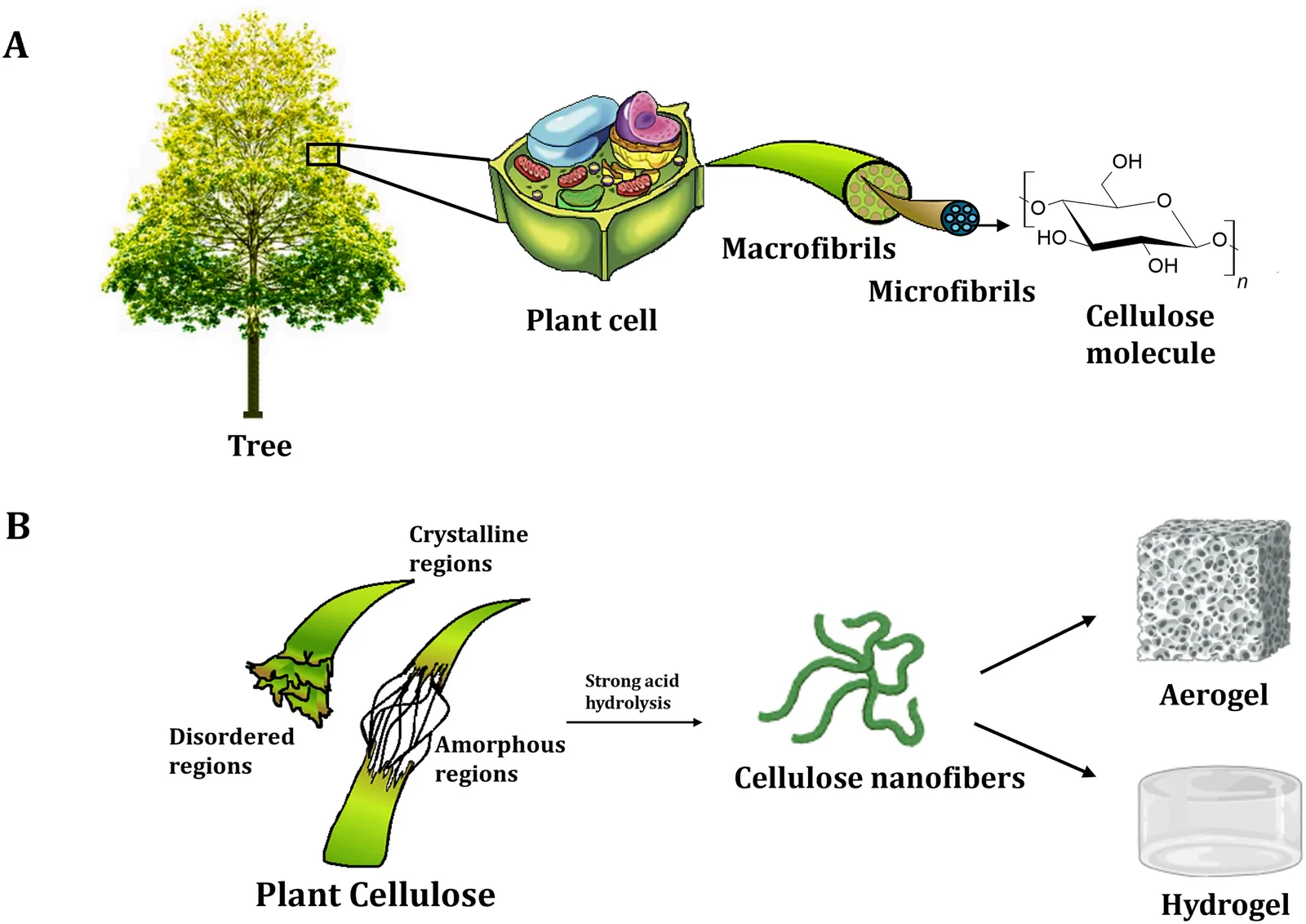
(A) Hierarchical organization of plant-derived cellulose. (B) Schematic diagram of the preparation of various nanocellulose forms (adapted from ref. 25 D. Miyashiro, R. Hamano and K. Umemura, Nanomaterials, 2020, **10**, 186, publisher MDPI).

Cellulose is mainly extracted from biomass using a variety of pretreatment methods, with the most common being chemical, physical, or combined.^26^ The most common types of pretreatments are presented in Table 1. Chemical pretreatments mainly include acid hydrolysis and alkaline-acid pretreatment. For example, acidic hydrolysis is used to produce cellulose nanocrystals.^27^ Physical pretreatments include high-pressure homogenization,^26^ microfluidization,^28^ cryocrushing,^29^ ultrasonication,^30,31^ grinding, and ball milling process,^32^ with ultrasonication being one of the most widely used. During the soaking of cellulose in deionized water, it is exposed to ultrasonic fibrillation (to isolate the nanofibers). The ultrasonication process can be carried out at different frequencies and power levels, depending on the required characteristics of the resulting material. In high-pressure homogenization, high shear forces produce defibrillated cellulose fibres.^26^ Combined pretreatment, which includes both chemical and physical pretreatments, is the most popular method.

**Table 1:** Cellulose pretreatment methods

Chemical	Physical	Combined
Acid hydrolysis	High-pressure homogenization	A combination of chemical and physical methods
Alkaline-acid pretreatment	Microfluidization	
	Cryocrushing	
	Ultrasonication	
	Grinding	
	Ball milling process	

The structure of cellulose is composed of a crystalline part (an inter- and intramolecular system of hydrogen bonds that are generated by the hydroxyl groups and free oxygen atoms) and an amorphous part. One of d-glucose's hydroxyl groups is extremely reactive and readily reacts with a variety of chemicals.^33^ Since cellulose has strong hydrogen bonds and a large molecular mass, it is not soluble in most solvents.^34^ Its hygroscopic features and lack of melting qualities limit its use in high-value-added applications.^35^

Cellulose can undergo many modifications to be exploited to its full potential across a variety of domains.^36^ The modification process can involve chemical reactions (sulphation,^37^ TEMPO-oxidation,^38^ etherification,^39^ silylation,^40^ and carbamylation^41^). The sulphation reaction, which introduces negatively charged sulphate esters onto the surface of NCC, is a noteworthy example of chemical modification.^37^ Usually, the oxidation of cellulose uses (2,2,6,6-tetramethylpiperidin-1-yl)oxyl (TEMPO) to convert hydroxymethyl groups to carboxyl groups while preserving secondary hydroxyl groups.^38^ Through the action of isocyanate, urethane bonds are formed *via* carbamylation, and thus the modified material is characterized by increased hydrophobicity.^41^ In this way, material with unique properties can be obtained. The variety of cellulose modifications is presented in Fig. 3.

**Fig. 3:**
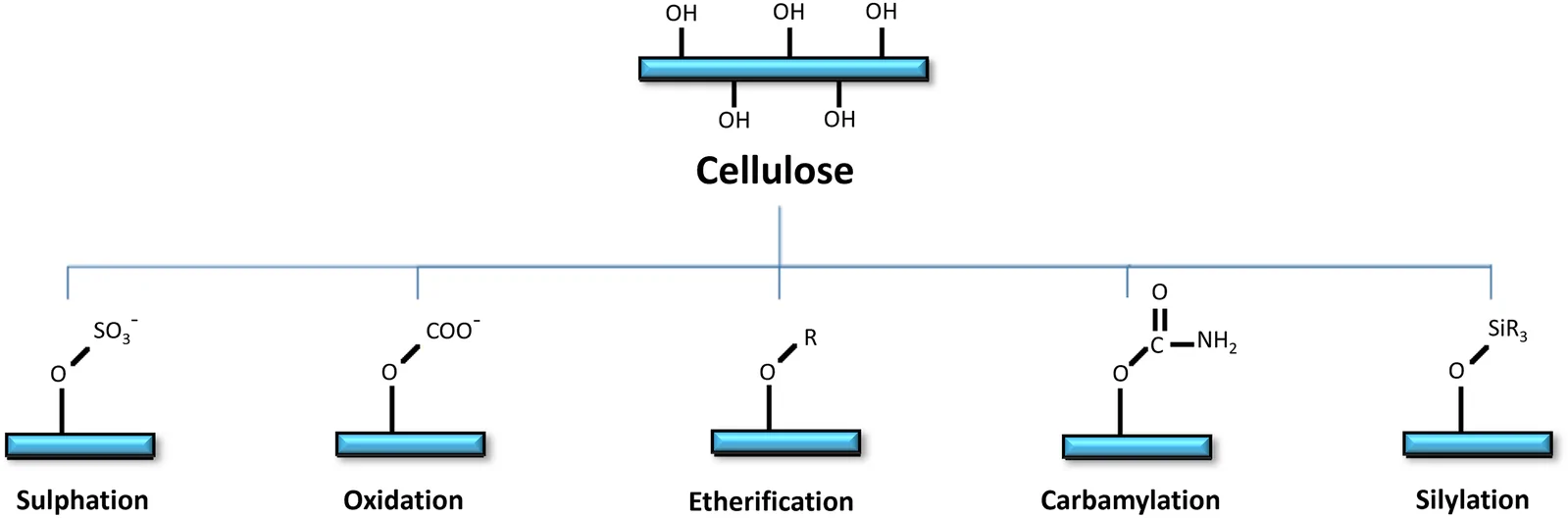
Modifications of cellulose.

The use of cellulose in the contemporary world is extremely wide, including various research applications and, mainly, industrial uses. Cellulose can be used in catalysis (as an ideal catalyst support matrix);^42^ adsorption of various chemical compounds (as an adsorbent);^43^ drug delivery (as drug carriers);^44^ pharmaceutical formulations (as stabilizers, enhancing bioavailability, stability, and affecting drug release profiles);^45^ food industry (as emulsion stabilizers);^46^ the papermaking industry (to improve tensile and other mechanical properties);^47^ electrophoresis of proteins (as supporting media)^48^ or tissue engineering (as reinforcement of biocomposites),^18^ as presented in Fig. 4.

**Fig. 4:**
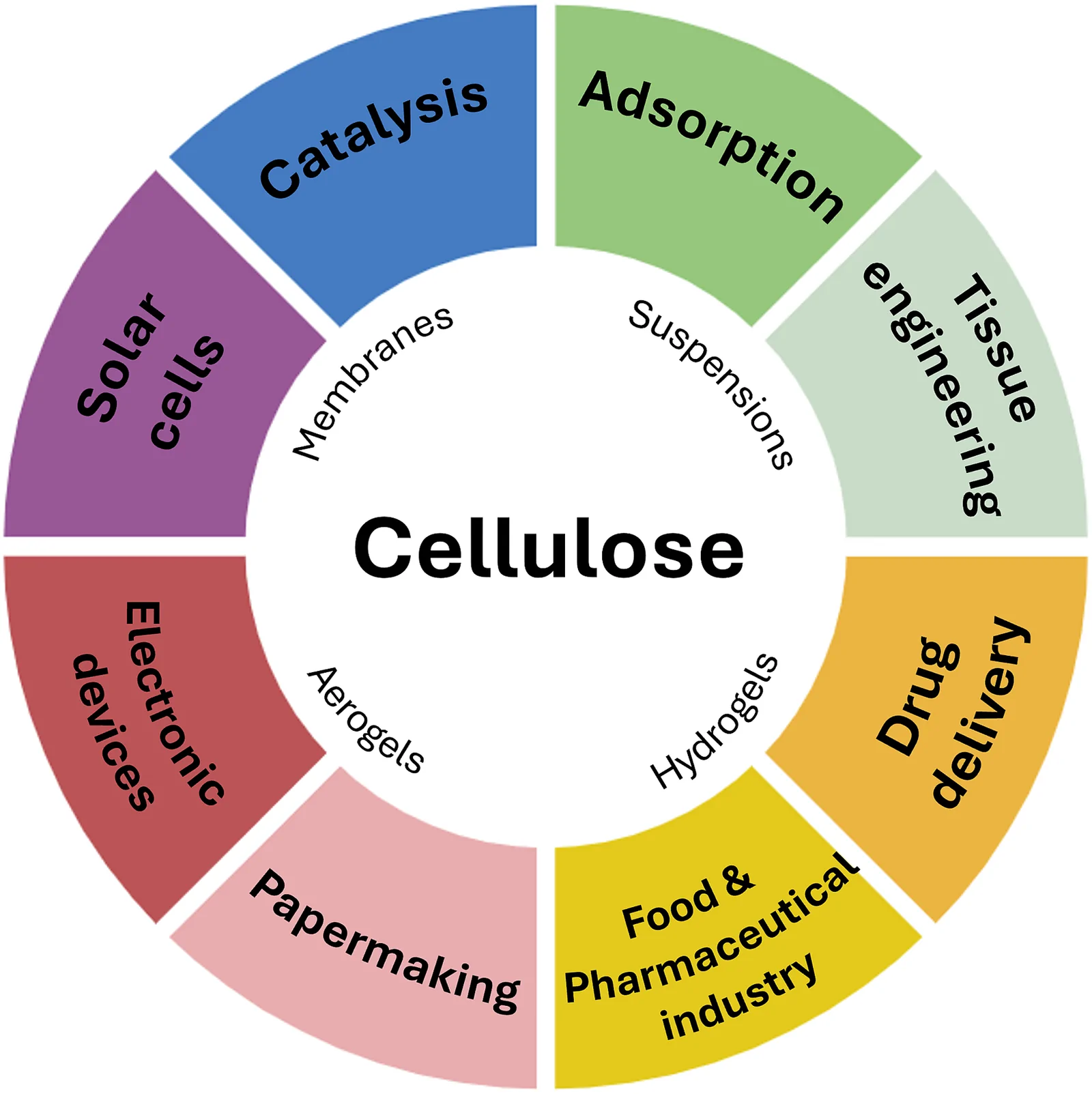
Applications of cellulose.

### Quaternary cellulose

To change the properties of cellulose, it is often functionalised; one example is the quaternization of cellulose with ammonium or phosphonium groups, followed by further modification into supported ionic liquids. The product of quaternization, quaternary cellulose, is a type of modification with a positively charged group *via* a free hydroxyl group in the chain backbone.^49^ It is highly biodegradable and non-toxic, among the many qualities that make it useful in water purification, the textile industry, papermaking, and the medical field.^50^ Thus, compared to unmodified cellulose, quaternary cellulose has improved water solubility, pH-stabilizing and flocculating properties, thermal stability, and redispersibility.^49,51^ These materials have many cationic groups on their surfaces, which facilitate the removal of selected chemical compounds (mainly anionic) through various interactions (*e.g.*, electrostatic, hydrogen-bonding, dispersion, and dipole–dipole interactions).^52^ Optimization of this process, including the NaOH-to-CHPTAC molar ratio, determines the extent of cellulose cationization.^53^ Surely, the cationization of cellulose notably increases the stability and dispersity of cellulose fibres in water.^54^ Furthermore, it can increase antibacterial activity due to the attachment of a surface-active alkyl chain, which typically confers bactericidal properties.^55^

Quaternization is one way to modify cellulose, creating a material with new and remarkable properties. To produce quaternary cellulose, 3-chloro-2-hydroxypropyltrimethylammonium chloride (CHPTAC) is primarily used; the cationization reaction is carried out under alkaline conditions. CHPTAC easily reacts with OH^−^ ions to obtain 2,3-epoxypropyltrimethylammonium chloride (EPTAC).^56^ In the next step, an alkoxide group is produced when the –OH group attached to C_6_ in some anhydroglucose units (AGU) reacts with a base (primarily NaOH). Cationic cellulose is formed when the alkoxide groups in cellulose react with EPTAC.^57^ It's crucial to keep in mind that EPTAC may react with water to form a diol, which can reduce the efficiency of quaternary cellulose formation. The amount of base is significant in the cationization process because it affects the degree of substitution; an excess of base promotes the hydrolysis of epoxide and polysaccharides.^58^ In addition to producing EPTAC from CHPTAC, a base also helps to weaken hydrogen bonds between molecules, increasing the chemical availability of cellulose.^59^ The quaternization process is shown in Fig. 5.

**Fig. 5:**
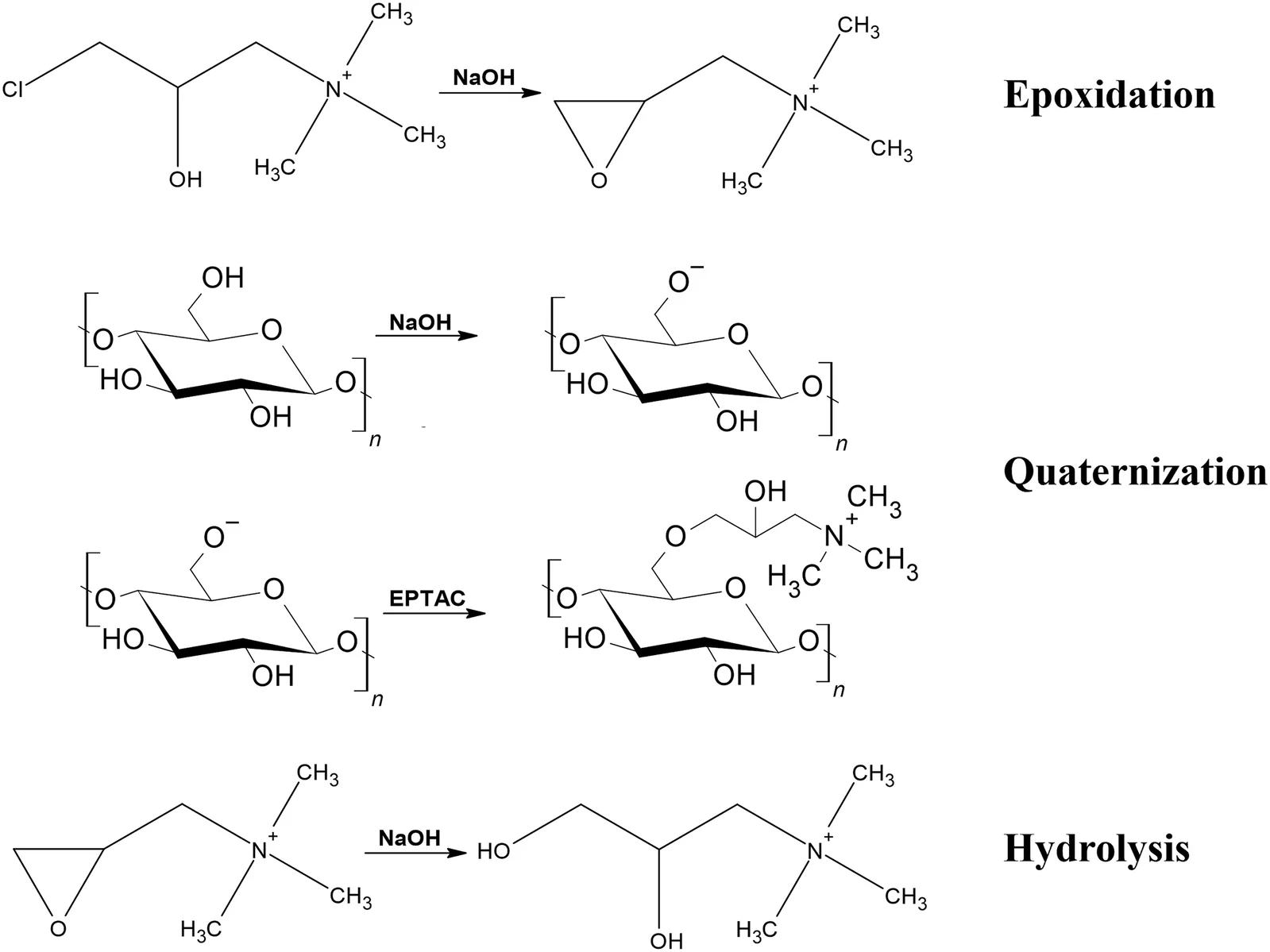
The most common preparation route of quaternary cellulose.

While CHPTAC is the most common reagent, cationization can also occur through a reaction between a tertiary amine (*e.g.*, *N*,*N*-dimethyldodecylamine; tetramethylethylenediamine; *N*,*N*-dimethyl-1-octadecylamine) and epichlorohydrin.^60^ Moreover, acrylamide, maleic anhydride, poly(allylamine hydrochloride),^61^*N*,*N*′-methylenebis-(acrylamide) (He *et al.*, 2014), and polyamide-epichlorohydrin resin^63^ are utilized to produce quaternary cellulose in the form of a hydrogel or an aerogel (in the case of aerogel, the material must be freeze-dried). A variety of quaternisation compounds possible to use in the cellulose quaternization process are presented in Table 2.

**Table 2:** Compounds used in the synthesis of quaternary cellulose

Name of the compound used in QACC synthesis	Structure
(3-Chloro-2-hydroxypropyl)trimethylammonium chloride	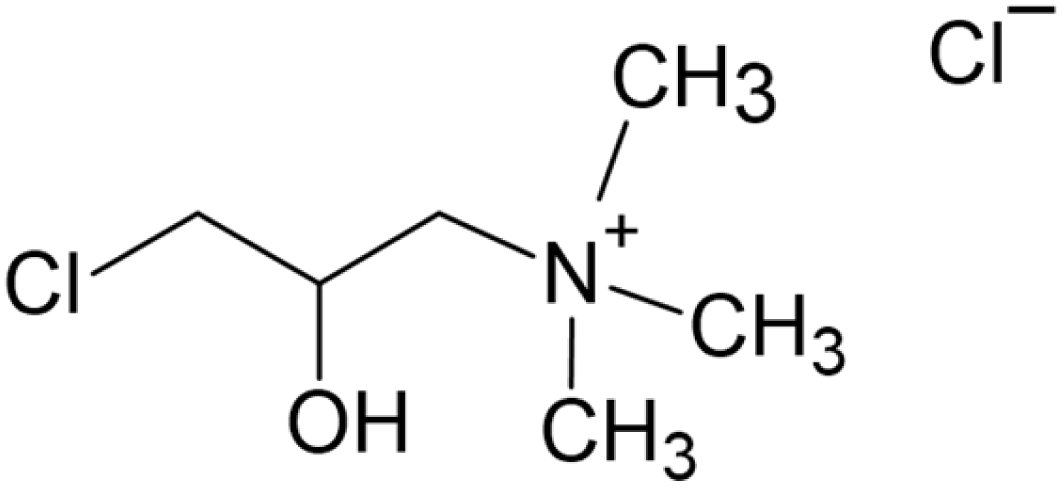
Glycidyltrimethylammonium chloride	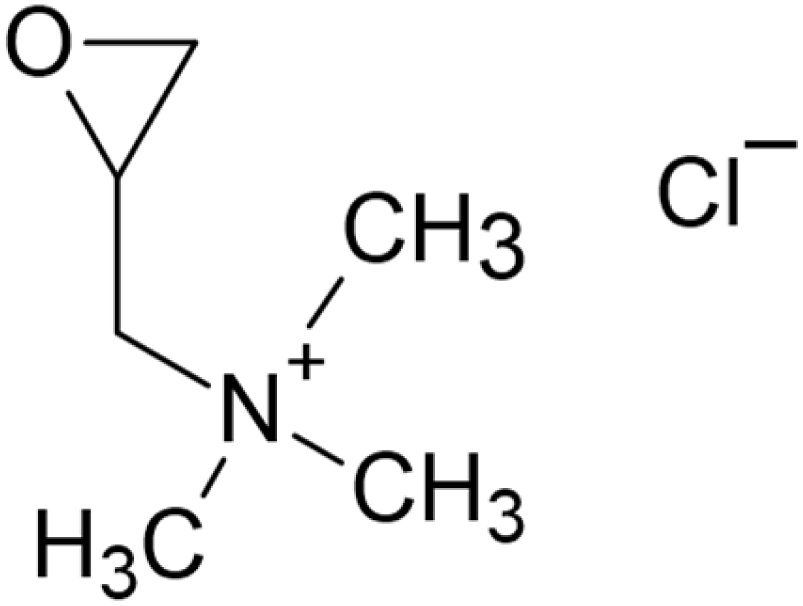
*N*,*N*-Dimethyldodecylamine	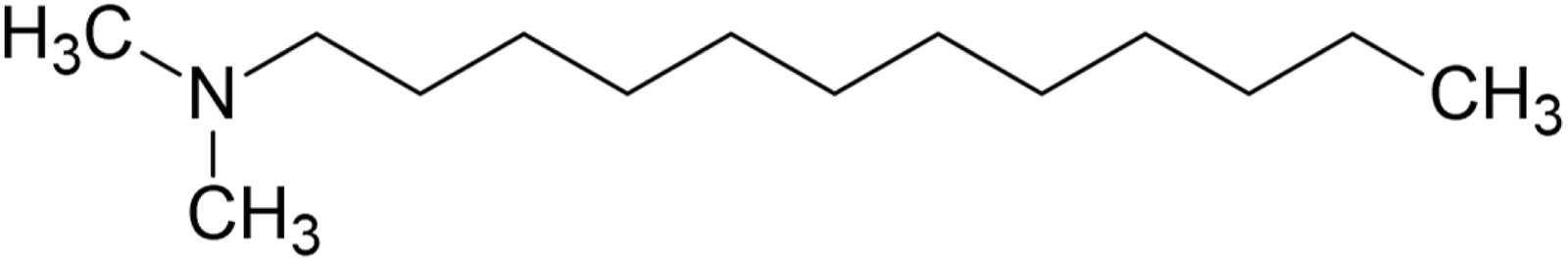
Epichlorohydrin	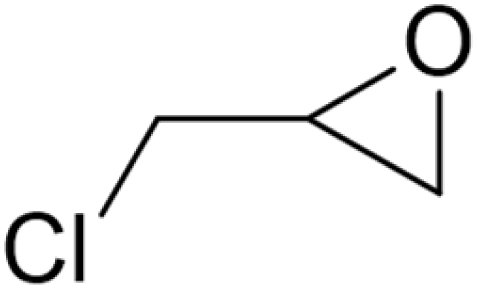
Tetramethylethylenediamine	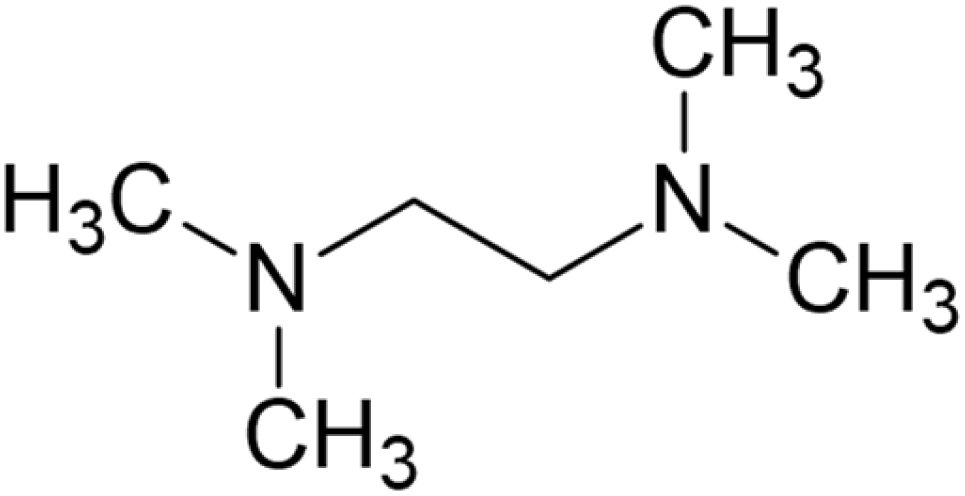
*N*,*N*-Dimethyl-1-octadecylamine	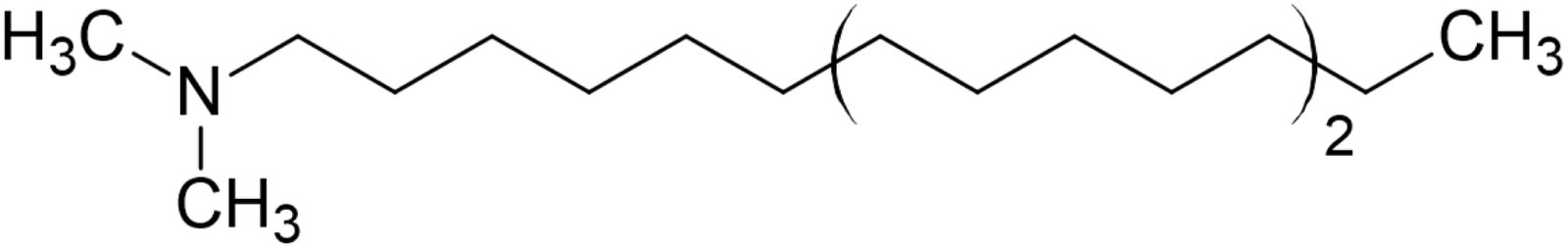
Tetraethylammonium acetate	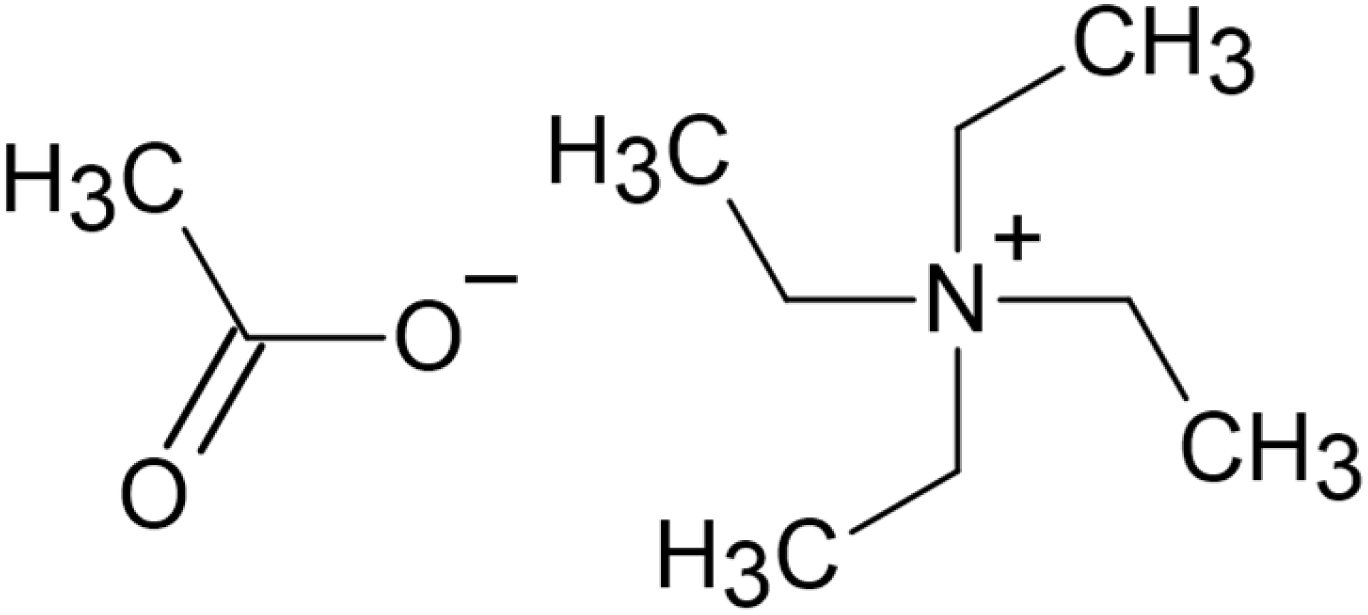
poly(allylamine hydrochloride) (PAH)	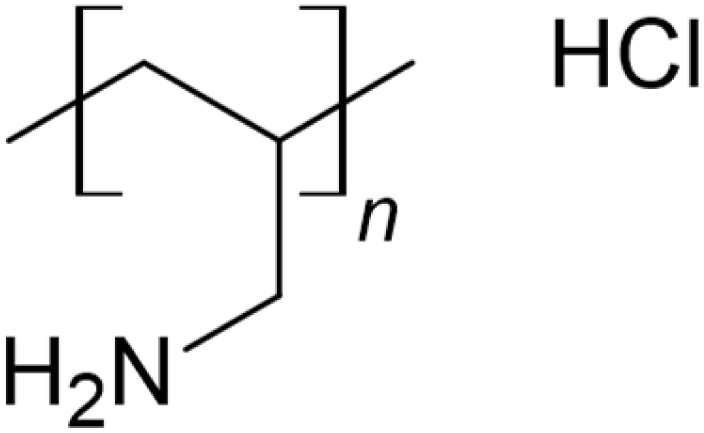
Acrylamide	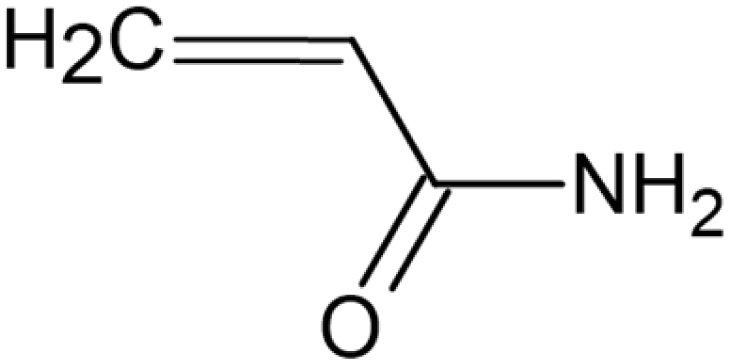
Maleic anhydride	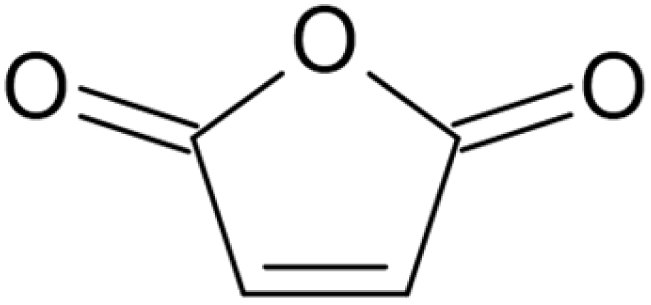
Triphenylphosphine	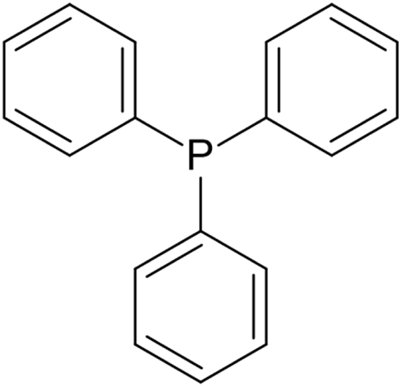

In cationization, one might also use quaternary phosphonium compounds, which have comparable properties.^64^ Xiong & Hu found that quaternary phosphonium salts are the subject of extensive research and are used as quaternizing agents in many extremely effective antibacterial materials. Due to the unique molecular structure of quaternary phosphonium cellulose, quaternary phosphonium salts, as cationizing agents, might also be used to cationize cellulose, thereby preparing a highly efficient adsorbent. Quaternary phosphonium cellulose can interact with negatively charged compounds (*e.g.*, anionic dyes).^65^

For instance, in the first stage of synthesis, cellulose reacts with epichlorohydrin under alkaline conditions. The excess epichlorohydrin is subsequently removed using, *e.g.*, isopropanol. Then, a triphenylphosphine/acetone solution is added. Afterward, the product is washed with ethanol to remove excess triphenylphosphine, and finally, after adjusting the pH to neutral (using 0.1 mol L^−1^ NaOH and 0.1 mol L^−1^ HCl), quaternary phosphonium cellulose is obtained.^65^

Although there are many strategies for preparing adsorbents based on quaternary cellulose, their advantages and disadvantages are not discussed systematically and are often addressed only indirectly or at the end of studies. This approach makes it difficult to understand the relationships among the synthesis method, the material's structure, and the final adsorption capacity. Direct functionalization (*e.g.*, using CHPTAC), grafting methods, cross-linking strategies, *etc.*, differ significantly in terms of reaction conditions, degree of functionalization, structural control, environmental impact, and process scalability.^66^ While simple and effective for introducing ammonium groups, it may encounter difficulties due to reaction conditions or limited control over structural uniformity. Grafting methods provide flexibility in designing the material's architecture; however, they entail increased synthesis complexity, reduced reproducibility, and high costs. Cross-linking improves mechanical durability and helps maintain three-dimensional structures; however, it may limit the availability of active sites and adversely affect adsorption processes.

The articles currently available do not adequately address the impact of synthesis methods on long-term durability, regenerative capacity, process scalability, and economic viability. It is therefore crucial to focus not only on the advantages but, above all, on the limitations of current synthesis methods. Over time, this may lead to higher yields of quaternary cellulose adsorbents and a focus on rational design.

### Environmental applications of quaternary cellulose

Because of its unique qualities and capacity for biodegradation and renewal, quaternary cellulose is utilized in the adsorption of hazardous chemicals. Adsorption is a simple, efficient, cost-effective, and environmentally benign technique for removing various chemical substances.^67^ Synergism between the adsorbent and the adsorbate is most important in the adsorption process. The more effectively an adsorbent is chosen, the more adsorbate molecules will be adsorbed.^68^ Adsorption optimization should be performed with consideration of all initial concentrations and pH values.^69^ Adsorption kinetics are crucial for assessing the effectiveness of adsorption. Based on kinetics, the time required to reach equilibrium and the correct kinetic model can be determined.^70^ On the other hand, adsorption isotherms are used to determine the type of adsorption process.^71^ Adsorption most often occurs due to electrostatic interactions, hydrogen bonding, competition from OH^−^ ions, and the structural features of the adsorbate and adsorbent. Most often, compounds in anionic form become adsorbed on quaternary ammonium cellulose, as presented in Fig. 6. All data discussed in the following section of adsorption studies concerning adsorption capacity, adsorption isotherms, and adsorption kinetics are presented in Tables 3–5.

**Fig. 6:**
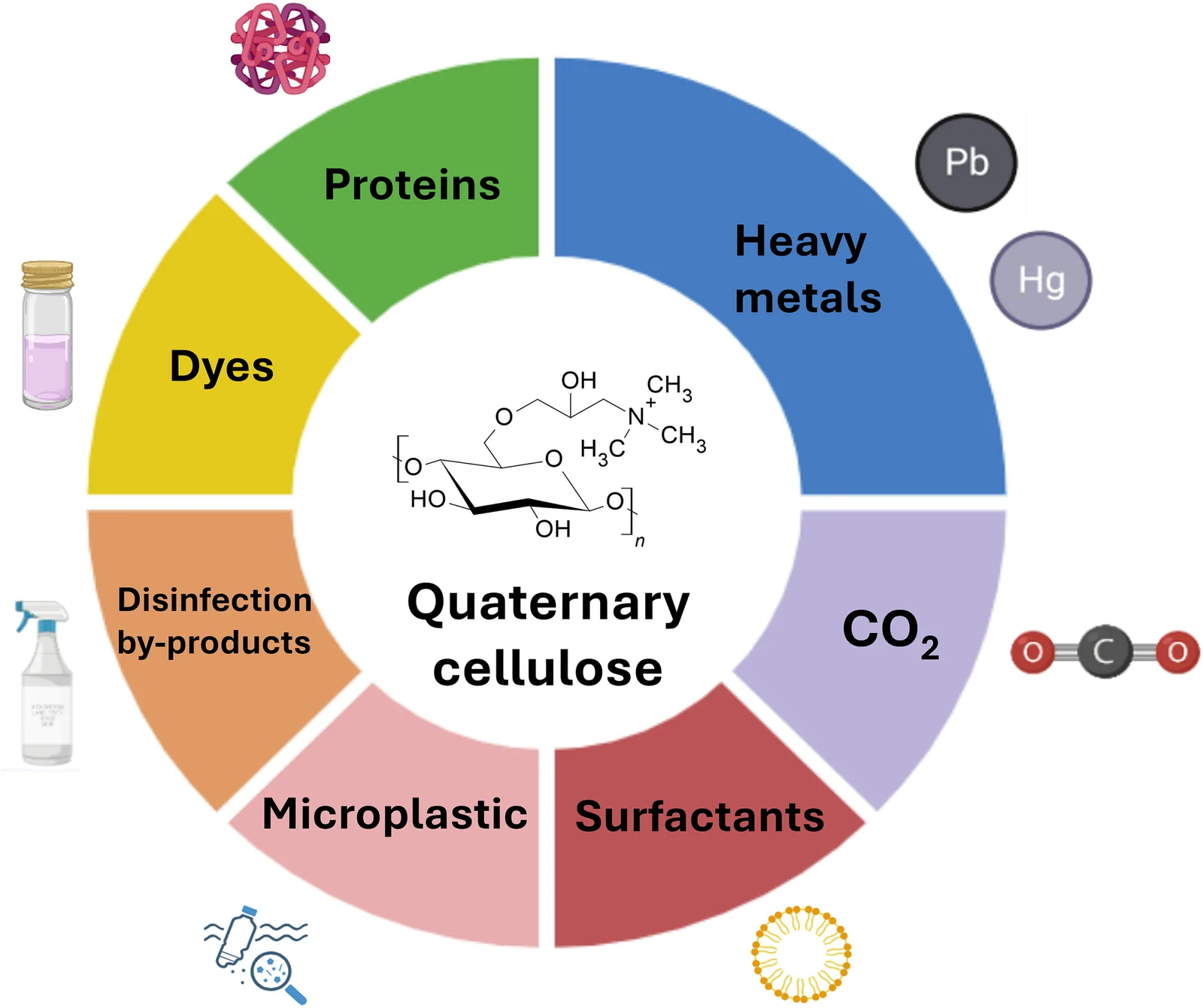
Pollutants removed by quaternary cellulose.

**Table 3:** Adsorption capacities of selected organic compounds

Name of hazardous materials	Capacity of adsorption (mg g^−1^)	Ref.
Eosin Y	717.00	61
	364.22	63
Congo red	304.34	77
	121.51	83
	314.00	79
Reactive blue 21	543.00	81
Acid black 24	332.80	65
Sunset yellow	107.08	82
SDS	32.50	84
Haemoglobin	0.088^a^	85
Myoglobin	0.423^a^	
Monochloroacetic acid	180.00	86
Dichloroacetic acid	251.00	
Trichloroacetic acid	350.00	
Cr(vi)	17.66	62
	33	87
CO_2_	0.18^a^	88
Au(i)	109.87	89
Copper(ii) ion	232.56	90
Sulphate ion	476.19	

a — Values given as mmol g^−1^.

**Table 4:** Langmuir and Freundlich isotherm parameters

Name of adsorbate	Details	Langmuir			Freundlich			Ref.
		*Q*_max_ (mg g^−1^)	*K*_L_ (L mg^−1^)	*R* ^2^	*K*_F_	*n*	*R* ^2^	
Eosin Y	44.85 °C	729.93	0.101	0.9999	25.35	6.15	0.6495	61
	Ambient temperature	364.22	0.783	0.9990	123.31 (mg L^−1^)	5.05	0.8720	63
Congo red	30 °C	314.00 ± 8	1.300 ± 0.3	0.9326	165.00 ± 8 (mg g^−1^)/(mg L^−1^)^1/*nF*^	0.15 ± 0.01	0.9669	79
	RHCC	102.35	0.042	0.9980	28.96 (mg^1−*n*^ L^*n*^ g^−1^)	5.06 (L mg^−1^)	0.9719	80
	EHCC	121.51	0.058	0.9995	31.75 (mg^1−*n*^ L^*n*^ g^−1^)	4.55 (L mg^−1^)	0.9702	
Reactive blue 21	50 °C	628.93	0.704	0.9991	344.80 (L g^−1^)	6.64	0.7158	81
Acid black 24	50 °C	332.80	0.020	0.9999	54.02 (mg^1−(1/*n*)^ g^−1^ L^1/*n*^)	3.23	0.9904	65
Sunset yellow	30 °C	107.08	2.232	0.9440	55.70	6.44	0.8710	82
SDS	60 °C	22.80	0.354	0.9790	4.89 (mg g^−1^)	35.09	0.6900	84
SDBS	44.85 °C	595.00	2.23 × 10^−2^	0.9952	90.00 (mg g^−1^)	3.28	0.9719	94
SLS		437.00	2.29 × 10^−2^	0.9973	168.00 (mg g^−1^)	7.25	0.9884	
SDS		392.00	1.49 × 10^−2^	0.9973	117.00 (mg g^−1^)	5.62	0.9713	
Cr(vi)	Ambient temperature	32.68	1.500	0.9900	22.69	14.38	0.8000	87
Au(i)	25 °C	109.87 ± 3.67	0.118	0.9887	26.38 ((mg g^−1^)(L mg^−1^)^1/*n*^)	3.60	0.9477	89
Copper(ii) ion	Ambient temperature	232.56	0.020	0.9970	15.35 (mg g^−1^)(mg L^−1^)^−1/*n*^	2.17	0.9669	90
Sulphate ion		476.19	0.008	0.9745	25.30 (mg g^−1^)(mg L^−1^)^−1/*n*^	2.26	0.9966	

**Table 5:** Parameters of pseudo-first-order and pseudo-second-order kinetic models

Name of adsorbate	Details	Pseudo-first-order kinetic model			Pseudo-second-order kinetic model			Ref.
		*k*_1_ (min^−1^)	*q*_e1_ (mg g^−1^)	*R* ^2^	*k*_2_ (g mg^−1^ min^−1^)	*q*_e2_ (mg g^−1^)	*R* ^2^	
Eosin Y	44.85 °C	8.475 × 10^−4^	15 997.42	0.9594	3.53 × 10^−4^	680.27	0.9998	61
	Ambient temperature	145.602 × 10^−3^	—	0.9650	8.13 × 10^−3^	—	0.9990	63
Congo red	30 °C	0.160 ± 0.02	59 ± 1.66	0.9317	0.003 ± 0.005	67 ± 1.8	0.9676	79
	RHCC	3.67 × 10^−4^	22.36	0.8855	0.044	85.91	0.9999	80
	EHCC	3 × 10^−4^	28.66	0.9031	0.038	94.77	0.9999	
Reactive blue 21	50 °C	0.007	171.00	0.7041	1.43 × 10^4^	617.00	0.9997	81
Acid black 24	50 °C	0.017	98.95	0.7960	0.005	112.13	0.9254	65
Sunset yellow	30 °C	1.794	65.32	0.9730	0.055	68.97	0.9980	82
SDS	60 °C	0.026	19.50	0.9950	0.0038	33.60	0.9960	84
SDBS	44.85 °C	3.70 × 10^−2^	513.00	0.9601	1.36 × 10^−4^	625.00	0.9971	94
SLS		3.68 × 10^−2^	347.00	0.9680	1.83 × 10^−4^	450.00	0.9944	
SDS		3.27 × 10^−2^	339.00	0.9214	1.66 × 10^−4^	379.00	0.9972	
Au(i)	Ambient temperature	1.790 ± 0.15^a^	70.79 ± 0.86	0.9824	0.045 ± 0.0033	72.46 ± 0.50	0.9951	89
Copper(ii) ion	Ambient temperature	0.056	150.13	0.9852	7.90 × 10^4^	163.90	0.9983	90
Sulphate ion		0.063	247.70	0.9369	5.90 × 10^4^	270.27	0.9970	

a Value given as L min^−1^.

### Adsorption of dyes

Dyes are widely used in the printing, cosmetics, and textile industries, and substantial quantities are eventually discharged into wastewater. Many dyes are carcinogenic,^72^ and hazardous to the environment (*e.g.*. dyes can contribute to soil degradation).^73^ Therefore, considerable efforts have been devoted to developing efficient, cost-effective, and environmentally friendly dye removal methods. One approach is to use quaternary cellulose structures that contain, for example, glycidyltrimethylammonium groups.^61^ As mentioned earlier, quaternary cellulose is usually employed in the form of hydrogels, aerogels, and solid materials in various shapes and sizes. Hydrogels are three-dimensional polymeric networks that are insoluble in water yet exhibit high water-retention capacity.^74^ Importantly, the adsorption capacity of quaternary cellulose can be further enhanced by employing it as a hydrogel. The semi-IPN hydrogel is a particularly promising example. Liu *et al.* described the preparation of such a hydrogel *via* a click reaction between poly(allylamine) hydrochloride (PAH) and a maleic anhydride-acrylamide copolymer (PAM) in water. Because PAH is a polymer with a large number of amine groups, it readily combines with a maleic anhydride-based copolymer, forming a hydrogel in water under mild conditions *via* the amino–anhydride reaction. Liu *et al.* used Eosin Y as a model adsorbate in adsorption studies. The authors analyzed the effect of adsorbent dosage on the adsorption process. Increasing the adsorbent dose from 5 to 10 mg initially enhanced the adsorption capacity; however, further increases in adsorbent mass decreased it. The highest adsorption capacity was observed at pH = 3 (1240 mg g^−1^). However, adsorption at near-neutral pH values may be more environmentally favourable, as it reduces the need for acid addition while still maintaining high adsorption performance. The high adsorption capacity observed under acidic conditions is due to electrostatic interactions between Eosin Y molecules and the hydrogel's protonated groups. Higher adsorption capacities result from electrostatic interactions between protonated, positively charged –OH and –NH_2_ groups at lower pH values. At alkaline pH, the –OH and –NH_2_ groups are deprotonated, substantially reducing the strength of interactions between the adsorbent and the dye and leading to much lower adsorption efficiency. Electrostatic interactions between the dye molecules and the quaternary ammonium cation contribute to adsorption.^75^ Ionic strength also plays a crucial role in adsorption, and increasing it gradually decreases the adsorption capacity. Adsorption of Eosin Y onto the semi-IPN hydrogel occurs *via* monolayer chemisorption (as indicated by the higher *R*^2^ of the Langmuir isotherm), involving the specific binding of dye molecules to active sites on the hydrogel surface. The adsorption kinetics are well described by the pseudo-second-order kinetic model, which assumes that the number of available active sites and the chemical interactions between the adsorbent and the adsorbate determine the adsorption rate. As active sites are occupied, the approach to equilibrium slows, although the initial stage of adsorption proceeds rapidly because dye molecules are quickly attracted to the positively charged ammonium groups. The three-dimensional, porous hydrogel facilitates the diffusion of dye molecules into its interior. According to the reported data, adsorption proceeded quickly, reaching equilibrium within 3 h and achieving an adsorption capacity of 717 mg g^−1^. The semi-IPN hydrogel is a better adsorbent than commercial adsorbents. It is characterized by higher adsorption capacity and a relatively short equilibrium time. However, no information was provided about adsorbent regeneration and reusability.^61^

However, C. Feng *et al.* reported an adsorption capacity of 364.22 mg g^−1^ for Eosin Y using quaternary cellulose aerogels. Notably, the adsorption process was largely independent of pH. C. Feng *et al.* also investigated the adsorption of mixed dye systems (Eosin Y and Methylene Blue). In this case, Eosin Y exhibited selective adsorption onto quaternary cellulose. Although cationization greatly enhances the adsorption of anionic dyes, it significantly reduces the adsorption of cationic dyes.^76^ The ammonium groups of the cellulose aerogel form positively charged adsorption sites that attract anionic dyes. The number of active sites increases with the degree of substitution, thereby enhancing the dye-attraction force and adsorption capacity. While the adsorbent's permanent positive charge ensures effective adsorption across different pH levels, the material's porous, three-dimensional structure promotes diffusion and interaction between the dye and the surface. The adsorption kinetics and isotherm data were comparable to those reported by Liu *et al.* The adsorption capacity decreased as the temperature increased (an exothermic reaction). The adsorption capacity was not significantly affected by the presence of chloride, sulphate, and carbonate ions in the wastewater. A major advantage of these adsorptive materials is their exceptional efficacy at continuously removing anionic dyes. Because the adsorption mechanism is primarily electrostatic, regeneration can be achieved by disrupting these interactions. Regeneration using HCl treatment maintained high adsorption capacities over the first five cycles, demonstrating promising reusability and economic benefits. This makes these materials a good choice as an environmentally friendly adsorbent for dyes.^63^

Congo red is another dye that can be readily removed by quaternary cellulose. D. Hu *et al.* investigated the adsorption of Congo red under various experimental conditions. At lower pH values, amino groups in the system become protonated. Adsorption capacity decreases with increasing pH. It increases slightly when the pH exceeds 7.68 (the point of zero charge). This behaviour may be attributed to hydrogen bonding between the dye's amino groups and the adsorbent's hydroxyl groups. Ion exchange between Congo red's –SO_3_^−^ groups and quaternary ammonium groups adsorbs dye molecules onto the adsorbent. Subsequently, unbound dye molecules can associate with previously adsorbed dye molecules *via* electrostatic interactions. The number of protonated amine groups decreases as pH increases, thereby reducing adsorption capacity. High ionic strength facilitates the adsorption of Congo red on quaternary cellulose by promoting dye aggregation. Monolayer chemisorption is supported by the Langmuir model and pseudo-second-order kinetics observed during the adsorption of anionic dyes on quaternary cellulose aerogel. Positively charged groups on the adsorbent rapidly interact with dye molecules, and the aerogel's porous, three-dimensional structure facilitates diffusion to active sites. As these sites are occupied, the adsorption rate slows and approaches equilibrium. The reported adsorption capacity of 304.34 mg g^−1^ is significantly higher than that of most commercial adsorbents.^77^ Similar results were reported by E. D. V. Giordano *et al.*, who obtained an adsorption capacity of 314 mg g^−1^. However, the authors indicated that the adsorption of Congo red is governed by several interactions, including ionic interactions between –NH_3_^+^ and –SO_3_^−^; Yoshida H-bonding between the –OH group of cellulose or the quaternary ammonium moiety and aromatic residues in the dye; and hydrogen bonding between hydroxyl groups from cellulose molecules and electronegative residues in the dye molecule.^78^ The proposed adsorption mechanism of Congo red on quaternary cellulose is shown in Fig. 7.

**Fig. 7:**
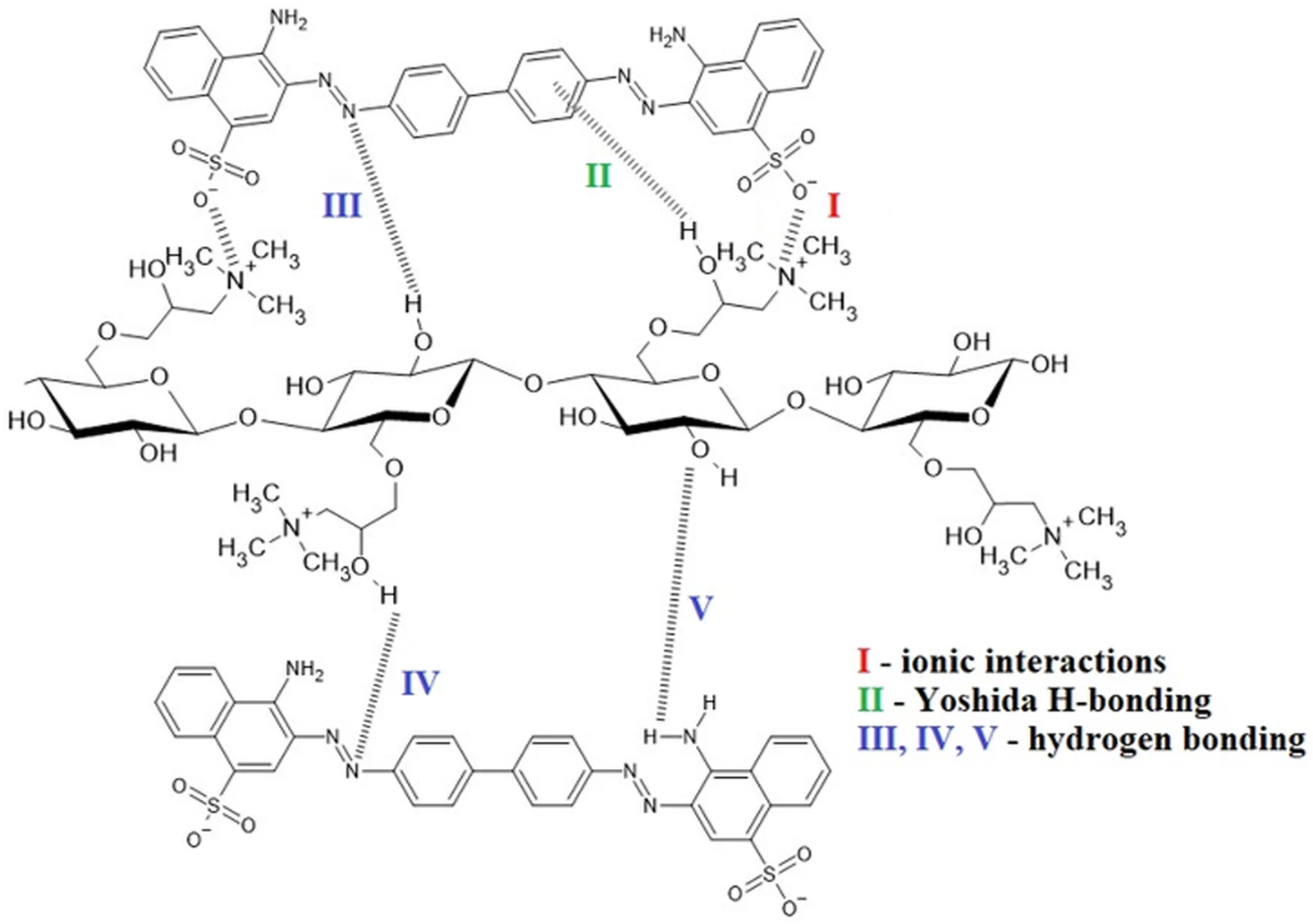
Schematic representation of the interactions between quaternary cellulose and the Congo red molecules. I – ionic interactions between –NH_3_^+^ and –SO_3_^−^; II – Yoshida H-bonding between the –OH group of cellulose or quaternary ammonium moiety and aromatic residue in dye; III, IV, V – bonding between hydroxyl groups from cellulose molecules and electronegative residues in the Congo red molecule.

Both unmodified cellulose and quaternary cellulose exhibit pseudo-second-order kinetics for Congo red adsorption. This suggests that chemisorption involving active sites on the adsorbent surface controls the adsorption rate. In accordance with strong, specific interactions at a given active site, the process starts quickly when dye molecules bind to available sites, then slows as sites become occupied. The Freundlich model indicates that the adsorption process is multilayered and heterogeneous. This model better explains the adsorption capacity of quaternary cellulose than that of unmodified cellulose, reflecting differences in energy and binding affinity across the adsorbent's active sites. The adsorption capacity improves noticeably (the process is endothermic) as the temperature rises because intramolecular interactions between the adsorbate and adsorbent are more favourable than interactions between the adsorbate and the solvent.^79^

P. S. Vassileva *et al.* investigated how the type of raw material used to prepare an adsorbent affected the ultimate value of adsorption capacity. The husks of rice and wheat were utilized to make quaternary cellulose. Due to electrostatic interactions between the dye and the cationic sites of the adsorbent, the adsorption of Congo red on quaternary cellulose derived from cereal by-products was highly dependent on pH, with the highest adsorption occurring under neutral or slightly alkaline conditions. According to adsorption studies, this process follows pseudo-second-order kinetics, with chemisorption being the rate-limiting step. Thermodynamic parameters suggested a spontaneous and favourable adsorption process, and isotherms indicate monolayer adsorption on a homogeneous surface. With an adsorption capacity almost 20 mg g^−1^ higher, the final adsorbent made from wheat husks proved to be superior. Both adsorbents have large adsorption capacities (102.35 and 121.51 mg g^−1^) that are noticeably greater than those of traditional cellulose, making them suitable for use as adsorbents. To investigate the possibility of reusing the adsorbents, sodium hydroxide solutions with concentrations of 0.01 M and 0.1 M were used. The data showed that the hydroxide concentration had a significant effect on the desorption efficiency. 0.1 M NaOH removed only 32–38% of the adsorbed dye, while 0.1 M NaOH gave a desorption efficiency of almost 77% for cellulose produced from rice husks and 70% for cellulose obtained from einkorn wheat husks. Due to the reversal of electrostatic interactions between the dye and quaternary cellulose, regeneration is possible and most effective in an alkaline environment (0.1 M NaOH).^80^

The removal of Reactive Blue 21 dye from quaternary cellulose was demonstrated by X. Zhang *et al.* The adsorbent was made utilizing tetramethylethylenediamine and epoxychloropropane rather than CHPTAC. Adsorption capacity drops dramatically from pH = 2 to pH = 7 and rises noticeably in an alkaline environment. Since there are many H+ ions in solution at lower pH levels, –SO_3_^−^ groups become protonated (–SO_3_H). The ≡NH^+^SO_3_^−^ is formed when the dye's sulphonic groups easily interact with quaternary ammonium groups. Deprotonation of –SO_3_H groups occurs at higher pH, reducing the number of contacts between the adsorbent and the dye. Dye molecules compete with an increasing concentration of OH^−^ ions. Thus, the adsorption capacity diminishes as pH rises. Keep in mind that R-21 is sensitive to high pH; in alkaline conditions, a portion of the dye containing vinyl sulphone groups changes into active vinyl sulphone groups. These groups can interact with cellulose's hydroxyl groups, thereby increasing adsorption efficiency. Furthermore, dye molecules aggregate more readily at high pH values, which facilitates adsorption. According to the pseudo-second-order kinetic model, adsorption occurs. The best way to characterize this adsorption is a Langmuir isotherm – it is a chemical monolayer adsorption onto a homogeneous adsorbent. R-21 adsorption is endothermic and spontaneous; at higher temperatures, adsorption was more effective. The adsorption capacity can reach 543 mg g^−1^, so quaternary cellulose can easily be used as an adsorbent for R-21 dye. Regeneration procedures and desorption results are not presented by the authors.^81^

Quaternary cellulose modified with (3-chloro-2-hydroxypropyl)trimethylammonium chloride was also used as a solid material to remove sunset yellow, an azo dye commonly used in the food industry for aesthetic purposes. The removal of sunset yellow was extremely fast, and at a measured concentration of 0.05 g/25 mL, it was nearly complete (99%) within the pH range 2–10. The sorption of sunset yellow followed a single-layer adsorption mechanism, as indicated by the Langmuir isotherm and a pseudo-second-order kinetic model. Importantly, the sorbent capability was also tested for binary solutions with either 10 mg L^−1^ or 100 mg L^−1^ of chloride, nitrate, phosphate, or sulphate, at pH 6.0, and the selectivity for the dye was still at the highest level. The sorbent was easily regenerated with 1.0 M NaOH or HCl to 95% and reused three times for sunset yellow removal with the same efficiency. Moreover, the authors presume that ionic and electrostatic forces dominate the interactions between the sorbent and the pollutant.^82^

Quaternary phosphonium cellulose can also be used to adsorb organic dyes. The adsorption of Acid Black 24 on quaternary phosphonium cellulose was investigated by Xiong *et al.* As the adsorbent dosage increases, dye adsorption increases because the number of active sites on the adsorbent increases. The ability for adsorption rises as pH rises. At pH = 7, the maximum adsorption capacity is achieved. Adsorption then decreases with further increases in pH. Acidic conditions protonate the SO_3_^−^ group to SO_3_H, resulting in weak interactions between SO_3_H and P^+^ and a low adsorption capacity. The number of SO_3_^−^ groups increases with pH, thereby improving electrostatic interactions and increasing adsorption capacity. Because OH^−^ ions compete with SO_3_^−^ groups under alkaline circumstances, adsorption is diminished. This is because there are more electrostatic interactions between OH^−^ and P^+^ than between SO_3_^−^ and P^+^ groups. Ionic strength is another important factor in adsorption. The adsorption capability increases with increasing ionic strength. Quaternary phosphonium cellulose has a high adsorption capacity that lasts even through the fifth cycle, making it reusable. Adsorption is primarily chemical and monolayer, following the Langmuir model and the pseudo-second-order kinetic model. It is evident from the thermodynamic study that adsorption is an endothermic, spontaneous process. Quaternary phosphonium cellulose is a promising adsorbent because its *q*_max_ (332.80 mg g^−1^) exceeds that of other adsorbents.^65^

Quaternary cellulose-based materials demonstrate outstanding potential as green adsorbents for dye-contaminated wastewater, primarily due to their tuneable surface chemistry and strong electrostatic interactions with anionic dyes. The studies presented clearly show that cationization enhances adsorption capacity, especially for sulphonated dyes, with adsorption efficiency strongly governed by pH-dependent protonation of functional groups. The examples presented showed that the adsorption processes generally follow pseudo-second-order kinetics and are well described by the Langmuir or Freundlich isotherms, indicating predominantly chemisorption. Moreover, the adsorption efficiency increases with increasing adsorption temperature. Still, such behaviour must be examined thoroughly, taking into account dye structure, aggregation processes, and the types of interactions. Selectivity toward anionic dyes is a great advantage, although it comes at the cost of reduced affinity for cationic dyes. Such behaviour clearly exhibits that electrostatic mechanisms of dye removal are predominant. The demonstrated tolerance to ionic strength and the use of waste-derived cellulose sources present quaternary cellulose-based adsorbents as not only environmentally but also economically viable for sustainable wastewater treatment.

### Adsorption of surfactants

Surfactants, found in many cosmetics and detergents, are compounds that enter wastewater in large quantities.^91^ Because of their unique structure, which consists of a hydrophobic tail and a hydrophilic head, surfactants exhibit an amphiphilic property.^83^ Surfactants cause many bubbles to form in water, which significantly hinders the flow of oxygen between water and air; this can ultimately lead to the death of aquatic organisms.^84,92^ An increasing amount of research is focusing on quaternary cellulose's ability to adsorb anionic surfactants.^93^

A study of SDS adsorption onto quaternary cellulose was conducted by Zou *et al.* using epichlorohydrin and an appropriate amine. The Williamson reaction was used to produce quaternary cellulose under alkaline conditions. Because of its positive charge, the quaternary ammonium cation (derived from, *e.g.*, *N*,*N*-dimethyldodecylamine) attracts the hydrophilic anion of anionic surfactants (such as sodium dodecyl sulphate and sodium dodecyl benzene sulphonate) through electrostatic interactions and van der Waals forces, which attract the hydrophobic functional group. Investigating how pH affected SDS adsorption, it was found that a pH of around 7 produced maximum adsorption. Adsorption effectiveness is impacted by the fact that, in acidic conditions, hydrogen ions outcompete quaternary ammonium ions, resulting in substantially weaker SDS-quaternary ammonium cation interactions. The combination of hydroxide ions with quaternary ammonium cations at pH > 7 causes a notable drop in adsorption. Temperature also has a major impact on surfactant adsorption. As the temperature increased, the adsorption capacity increased, reaching a maximum at 60 °C. Beyond this temperature, the adsorption capacity decreased due to high-temperature desorption of SDS adsorbed by intramolecular van der Waals forces. The adsorption process of SDS on quaternary cellulose occurs quite rapidly (within 3 h). The *R*^2^ values obtained during pseudo-first-order and pseudo-second-order kinetic studies are very close, with a slightly higher value for the pseudo-second-order model; therefore, the adsorption reaction is best described by this model (indicating chemical adsorption). The isotherm that best describes the adsorption of SDS on quaternary cellulose is the Langmuir isotherm (monolayer adsorption occurs). The process is spontaneous and endothermic, according to thermodynamic research. The maximum adsorption capacity is 32.5 mg g^−1^. It should be noted that quaternary cellulose can be employed as an adsorbent for anionic surfactants due to its biocompatibility and straightforward synthesis.^84^

Zhang *et al.* investigated the adsorption process not only for SDS but also for SLS (with a lauryl alkyl chain, meaning it is derived from a natural mixture of alkyl chains between 8 and 18 carbon atoms) and SDBS. During cellulose quaternization, they employed CHPTAC directly. A comparison of the two studies' findings shows that adsorption is significantly affected by the method used to obtain the adsorbent. The adsorption maximum for SLS in this instance occurred at a pH of around 6, while the maximum for SDBS and SDS occurred at a pH of around 5. From the study of adsorption kinetics, the *R*^2^ values of the pseudo-first-order model are typically substantially lower than those of the pseudo-second-order model. The Langmuir isotherm characterizes the adsorption of all three surfactants, indicating that adsorption is monolayer and primarily chemical. Thermodynamic studies also indicate that it occurs endothermically, spontaneously, and with an increase in entropy. During adsorption, chemical bonds were formed between the Lewis bases of the anionic surfactants and the quaternary ammonium cations. The adsorption capacity at 318 K is 610 mg g^−1^ for SDBS, 441 mg g^−1^ for SLS, and 379 mg g^−1^ for SDS.^94^

Electrostatic interactions between cationic ammonium groups and anionic parts of surfactants constitute the primary mechanism for the adsorption of anionic surfactants onto the surface of quaternary cellulose. The availability of active sites and the diffusion of surfactants depend on the material's shape and porosity. Adsorption is further stabilized by secondary hydrophobic interactions between cellulose and surfactant tails. The surface area of the adsorbent, its structural accessibility, and the density of functional groups affect the adsorption efficiency. The above studies do not describe the regeneration of quaternary cellulose following the adsorption of surfactants such as SDS, SLS, or SDBS.

The amphiphilic nature and persistence of anionic surfactants in wastewater pose a significant ecological risk, and their removal presents a considerable challenge. In the case of quaternary cellulose, the adsorption of anionic surfactants is driven by synergistic electrostatic interactions between quaternary ammonium groups and anionic head groups, as well as van der Waals interactions involving the hydrophobic alkyl chains. As with dyes, adsorption efficiency is strongly pH-dependent, with both acidic and alkaline conditions reducing efficiency due to competitive interactions with H^+^ or OH^−^ ions. Studies also indicate that temperature generally enhances adsorption up to an optimum, demonstrating that the balance between adsorption forces and surfactant mobility must be optimized. Kinetic studies reveal the formation of monolayers, indicating that chemisorption is the rate-limiting step in this process. Importantly, adsorption capacity is highly sensitive to the quaternization method and surfactant structure, emphasizing the critical role of synthesis strategy in adsorbent performance.

### Adsorption of disinfection by-products

Disinfection by-products are hazardous substances that can be found in drinking water. They are harmful to both the environment and human health; for example, chlorinated acetic acids are carcinogenic.^95^ These compounds are formed through reactions between disinfectants and natural organic compounds during the disinfection of drinking water.^96^ Shi *et al.* employed quaternary ammonium cellulose (using CHPTAC as a cationizing agent) as an adsorbent to remove three chlorinated acetic acids: mono-, di-, and trichloroacetic acid. The primary adsorption mechanism involves electrostatic interactions between the cationic groups of the adsorbent and the carboxylate groups of the acids. Mass transfer depends on surface area, porosity, and polymer swelling, while the number and accessibility of active sites depend on the degree of substitution. Additionally, hydrogen bonding and hydrophobicity contribute to enhanced stability. Ionic strength and pH affect the adsorption of chlorinated acetic acids and their competition with other anions present in solution. The adsorption capacity is strongly pH-dependent: for dichloroacetic acid (DCAA), it was twice as high under strongly acidic conditions compared to chloroacetic acid (MCAA) and trichloroacetic acid (TCAA). The maximum adsorption capacity for DCAA occurs at pH = 4, whereas for MCAA and TCAA, it is at pH = 5, beyond which the adsorption capacity declines sharply. At pH values below 3, hydrogen ions inhibit the ionization of chlorinated acetic acids, thereby reducing anion concentration and lowering the adsorption capacity. Above pH = 5, increasing the concentration of hydroxide ions competes with the quaternary ammonium groups, significantly decreasing adsorption.^97^ The adsorption kinetics of three chlorinated acetic acids follow a pseudo-second-order model, indicating that chemical sorption at active sites regulates the adsorption rate. Higher temperatures (*e.g.*, 318 K) enhance adsorption efficiency, demonstrating that the adsorption is endothermic. Adsorption is a spontaneous and entropy-increasing process. The Langmuir isotherm model fits the data better than the Freundlich model, suggesting that adsorption occurs primarily as a chemical monolayer on a homogeneous surface. At 318 K, the maximum adsorption capacities for MCAA, DCAA, and TCAA are 180, 251, and 350 mg g^−1^, respectively, with TCAA typically exhibiting the highest capacity due to stronger interactions with cationic sites. Adsorption onto quaternary cellulose occurs rapidly and more efficiently than with commercial adsorbents. In addition to being a non-toxic biosorbent, quaternary cellulose can be regenerated using sulphuric acid (VI) and recycled. Its adsorption capacity after six recycling cycles decreases only slightly compared to the original capacity. Therefore, quaternary cellulose can be employed as an effective adsorbent for the removal of chlorinated acetic acids from drinking water. The structural stability of the cellulose network, combined with its cationic groups, helps explain why bound anions are successfully released during desorption, while the material retains its functionality.^86^ Adsorption capacities for selected compounds are presented in Fig. 8.

**Fig. 8:**
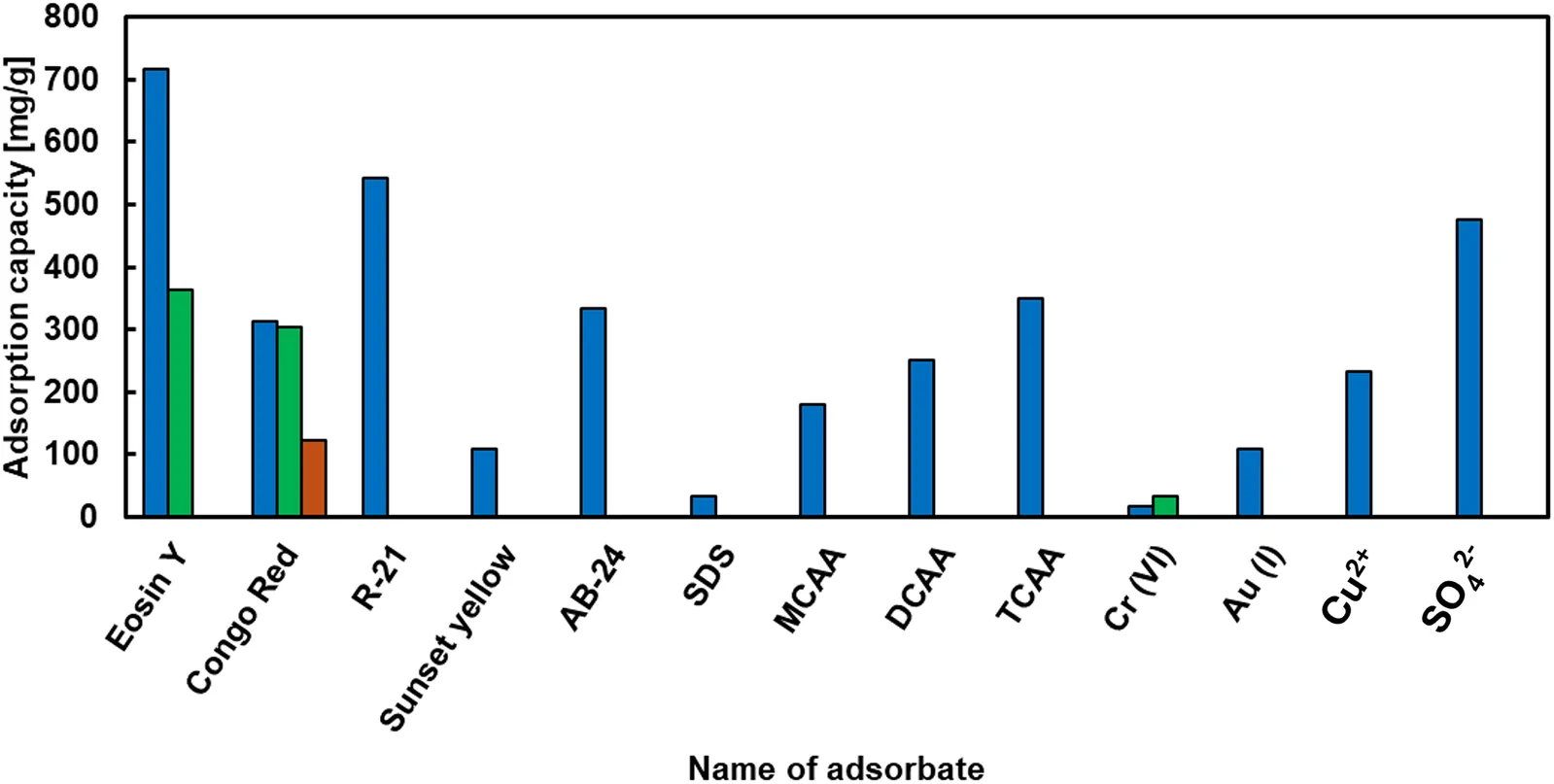
Adsorption capacities of selected pollutants.

In the removal of chlorinated acetic acids, adsorption is highly pH-dependent, with optimal results under slightly acidic conditions. Since these disinfection by-products are anionic in nature, their characteristics resemble those of dyes and surfactants, and the adsorption process exhibits similar kinetic behaviour. Chemisorption predominates, occurring rapidly and spontaneously, with efficiency improving as the temperature rises.

### Adsorption of proteins

Notable research has also been done on the use of quaternary cellulose for the adsorption of variety of biomacromolecules, including proteins.^98–100^ Protein adsorption onto quaternized cellulose is usually controlled by the relationship between solution pH and the isoelectric point of the protein. At pH values above the isoelectric point, proteins acquire a global negative charge and are able to interact electrostatically with the cationic groups of the adsorbent. Below the isoelectric point, this attraction is weakened or may be replaced by electrostatic repulsion. However, protein uptake is also influenced by the distribution of charged domains on the protein surface, the degree of substitution in quaternary cellulose, and the accessibility of adsorption sites which might be connected to adsorbent porosity.^99^ Most studies on the adsorption of those biomacromolecules focus on a limited number of model proteins, particularly bovine serum albumin, haemoglobin, and myoglobin. In case of quaternary cellulose, both plant-derived and bacterial cellulose sources have been used, usually using simple trimethylammonium groups as cationizing element. These systems are generally selected to examine how protein charge, solution pH, and cellulose morphology influence the adsorption process.

Niide *et al.* state that quaternary bacterial cellulose is an effective protein adsorbent for, *e.g.* haemoglobin or myoglobin. The cationization process of bacterial cellulose occurs similarly to that of plant cellulose, involving CHPTAC. The effect of pH on haemoglobin adsorption is crucial. The maximum adsorption capacity for haemoglobin on quaternary bacterial cellulose is reached at pH = 10, with adsorption capacity increasing as pH rises. pH below the isoelectric point, which for haemoglobin is equal to 6.8, occurs with a very low efficiency. Consequently, electrostatic interactions facilitate the adsorption of haemoglobin onto the adsorbent. The adsorption capacities of quaternary bacterial cellulose produced using dimethyl sulphoxide and dimethylformamide as solvents are the highest. Quaternary bacterial cellulose as an adsorbent is characterized by high selectivity; under the same conditions, adsorption of thymol blue occurs with much lower efficiency than the adsorption of proteins, *e.g.* haemoglobin. This is an effect of the distinct structure of quaternary bacterial cellulose compared to quaternary plant cellulose: its microfibrils are significantly thinner and ribbon-like, yet retain their fine structure. Importantly, the protein adsorption on quaternary bacterial-based cellulose is higher than on adequate plant-based cellulose under identical synthesis conditions, emphasizing the importance of intrinsic structure in adsorption studies. Quaternary bacterial cellulose has an adsorption capacity of 88.0 × 10^−3^ mmol g^−1^ for haemoglobin and 423 × 10^−3^ mmol g^−1^ for myoglobin.^85^ This specific structure of quaternary bacterial cellulose, namely porosity combined with nanofibrous form, along with the presence of cationic ammonium groups, it a favourable adsorbent for proteins, especially large number of active sites for electrostatic interaction with negatively charged protein molecules. The number and availability of active sites depend on the degree of quaternization. Protein diffusion is facilitated by an open network with a large surface area. Electrostatic interactions underlie this type of adsorption, and the adsorption capacity depends on the density and morphology of the active sites.^101^

Recently, quaternized cellulose microspheres have also been used to adsorb bovine serum albumin (BSA). The adsorption mechanism is similar to that described in the above. According to the pseudo-second-order kinetic model, proteins adsorb onto hierarchically porous microspheres of quaternary cellulose, indicating chemisorption through ionic interactions. The attraction between negatively charged protein domains and positively charged ammonium groups initially causes rapid adsorption, which then slows down as sites are occupied and equilibrium is reached. The hierarchical structure improves the accessibility of active sites and adsorption capacity, and the isothermal data fit the Langmuir model, indicating monolayer adsorption. Moreover, the porous structure of the cellulose microspheres enabled rapid mass transfer and excellent adsorption performance, achieving a maximum adsorption capacity of 108.6 mg g^−1^ of BSA within 90 min, with enhanced selectivity at pH 6.0 and reusability, maintaining 80% adsorption efficiency after 5 adsorption–desorption cycles. Importantly again, the adsorption is proceeded above the isoelectric point for BSA (4.6–5.8). This shows the microspheres' structural stability and efficient regeneration of active binding sites upon repeated use.^100^

Quaternary cellulose, derived not only from plants but also from bacteria, exhibits strong potential for selective protein adsorption, especially due to its positively charged surface and nanofibrillar structure. Protein adsorption is regulated by electrostatic interactions at alkaline pH values and above its isoelectric point. Superior adsorption capacities and selectivity for proteins are especially observed for nanofibrillated quaternary structures.

### Adsorption of heavy metals

It is possible to adsorb heavy metals such as gold and chromium using quaternary cellulose. The two main ways that chromate and dichromate ions can pollute the environment are through volcanic activity and effluents from the nuclear and timber industries, among other sources. Chromium compounds are carcinogenic to humans at high concentrations and toxic to other living things.^68^

He *et al.* devised a technique for adsorbing Cr(vi) compounds using quaternary cellulose aerogels. In this instance, CHPTAC was reacted with cellulose nanofibres, and the resulting material was lyophilized to form an aerogel. Quaternary ammonium groups interact electrostatically, exhibiting affinity for oxyanions. The adsorption of Cr(vi) ions on quaternary cellulose in the form of an aerogel is mainly an electrostatic process. In aqueous solutions, Cr(vi) occurs as anions, which are strongly attracted to the positively charged cationic groups of quaternary cellulose. The positive charge on the aerogel surface promotes effective interactions with Cr(vi) anions. Covalent cross-linking of the aerogel was used to strengthen its three-dimensional structure. The resulting aerogel has a large specific surface area, high porosity, and low density, all of which improve Cr(vi) removal from water. At pH = 3, the maximum adsorption capacity is reached. The predominant ions at this pH are hydrogen chromate ions, which can exchange with a Cl^−^ ion from the quaternary cellulose. The predominant form, H_2_CrO_4_, decreases Cr(vi) adsorption at pH < 3. Chromate and dichromate ions predominate in solution at higher pH values, and their ion exchange requires two chloride ions. Additionally, as OH^−^ ions accumulate in an alkaline environment, they compete with chromium ions, thereby reducing adsorption effectiveness. The adsorption process occurs very quickly, as almost 100% of Cr(vi) was removed after just 50 minutes. The Langmuir isotherm best describes the process; the maximum capacity is 17.66 mg g^−1^. The adsorption time and capacity are much higher than those of previous adsorbents. The adsorbent can be used multiple times for adsorption (after four adsorption cycles, the adsorption percentage decreases slightly), and the adsorption process is exothermic (as temperature increases, adsorption capacity decreases).^16^

A technique for effectively removing Cr(vi) from mine waste-contaminated waters was developed by Etale *et al.* They pointed out that, in this instance, preliminary removal of Fe and Al is essential for effective adsorption to take place because, in the absence of such elimination, ions will compete with one another for active sites on the adsorbent surface. Quaternary cellulose was synthesized using GTMAC. The mechanism of adsorption is described in the paragraph above. The most efficient pH for the adsorption process was 4. This process is compatible with second-order kinetics (chemical sorption was the limiting step). The adsorption of Cr(vi) ions is multilayered and involves energetically heterogeneous sites, according to the very good results obtained for the Freundlich and Dubinin–Radushkevich adsorption isotherms (energetically favourable chemisorptive interactions). There is no data about regeneration in this article.^102^ In Fig. 9, optimal pH values for selected compounds are presented.

**Fig. 9:**
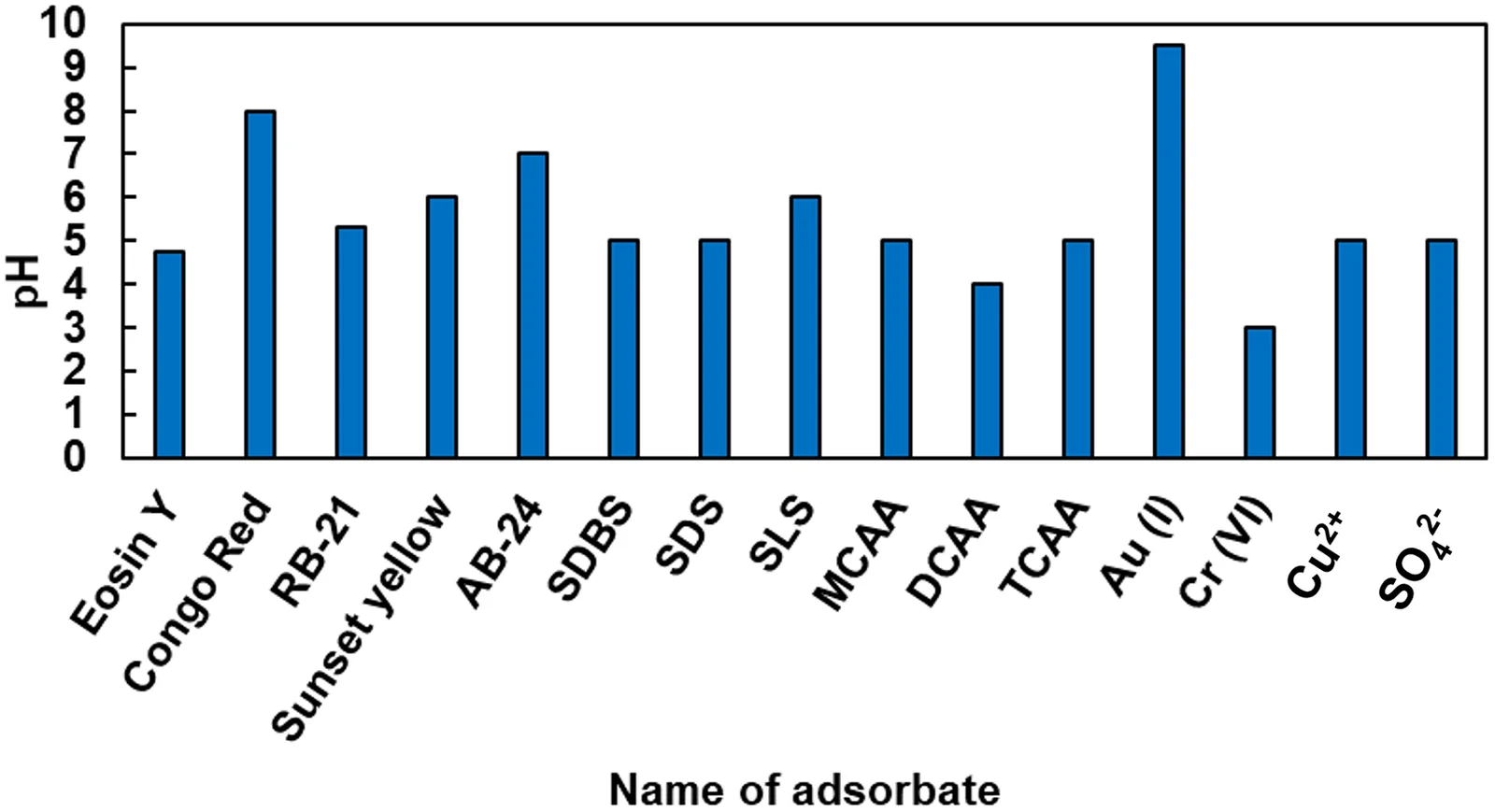
Optimal pH values for the adsorption of selected pollutants.

On the other hand, Sarıoğlu *et al.* used CHPTAC in the quaternary cellulose adsorption procedure and found that adsorption occurs more effectively at pH 6–10. Stronger interactions between Cr(vi) anions and quaternary cellulose are caused by the conversion of hydrogen chromate ion and chromate ion, and the decreased concentration of chloride ions in solution. The maximal adsorption capacity is 32.68 mg g^−1^, and the adsorption process is exothermic, fast (3 minutes), and follows the Langmuir isotherm, indicating monolayer adsorption. It was also studied what happens when chloride, nitrate, and sulphate ions are added to the solution. The adsorption of Cr(vi) decreases with increasing concentration in solution; for sulphate ions, this is particularly noticeable when the rate of Cr(vi) removal decreases. The adsorbent obtained by Sarıoğlu *et al.* exhibits extremely high adsorption capacity and can be reused multiple times. Regeneration of the adsorbent is carried out using 1 M HCl or H_2_SO_4_. Quaternary cellulose can be utilized again in subsequent adsorption cycles. Research data indicate that even after three recovery cycles, adsorption efficiency remains high, demonstrating the adsorbent's mechanical and chemical durability. Its practical value in environmental applications is enhanced by the ease of use and speed of the regeneration process.^87^

Due to its many benefits, including its catalytic properties, high thermal conductivity, and chemical durability, gold is one of the noble metals used in jewellery and industry. Recovery of gold from secondary resources, such as electronic waste or spent catalysts, can be more efficient and environmentally benign than recovery from ores. Finding a solvent that will dissolve gold, however, is difficult. To circumvent this, cyanide leaching is performed in an alkaline medium (this procedure does not emit HCN). Very poor adsorption capacities were observed in previously investigated adsorbents, such as activated carbons and graphene-based materials. A technique for gold adsorption utilizing quaternary cellulose (obtained using CHPTAC) was developed by X. Lin *et al.* Strong electrostatic interactions between cellulose cationic groups and the negative Au(CN)_2_^−^ complex in an alkaline solution are the main mechanism of adsorption. Ammonium groups enable rapid and precise capture of Au(i). In addition, the fibrous structure of the adsorbent promotes rapid, effective adsorption due to its large specific surface area and facilitated diffusion of ions to active sites. The surface of quaternary cellulose is positively charged at pH values lower than pH_pzc_ and becomes negatively charged when pH = 9.76 is exceeded. The pH_pzc_ value is calculated to be 9.76. Owing to HCN's toxicity (p*K*_a_ = 9.21), adsorption experiments were conducted between pH 9.5 and 12. The adsorption capacity decreases as pH rises. This is due to repulsion on the cellulose surface at higher pH levels, which eventually reduces the number of binding sites available for Au(i) adsorption. Furthermore, an increase in pH causes an increase in the quantity of OH^−^ ions, which compete with Au(i) ions. Thus, the best adsorption values are obtained at pH = 9.5. According to kinetic studies, adsorption proceeds rapidly, reaching equilibrium in about five minutes. This suggests that the adsorbent's active sites effectively bind anions and that mass transfer occurs at a very rapid rate. Adsorption can be physical or chemical, as indicated by the values of the pseudo-first- and pseudo-second-order models. The adsorption process is better described by the Langmuir model, indicating monolayer coverage on homogeneous active sites. Cl^−^ ions from CHPTAC are exchanged for Au(CN)_2_^−^ ions throughout the adsorption process. The high gold recovery rate in the presence of extra copper(ii) ion contamination in the water shows how selective the adsorption process is. The biosorbent obtained by Lin *et al.* had an adsorption capacity higher than that of previously examined activated carbons. Quaternary cellulose has a high adsorption capacity (109.87 mg g^−1^) and a high adsorption rate, making it an effective tool for gold recovery.^89^

Pollution by transition-metal cations and inorganic anions is emerging as a major issue due to the increasing volumes of various post-industrial contaminants entering the water supply. Sulphates are quite common; they may be found in natural waterways. An excessive concentration of them can cause the water to become mineralized, steel pipes to corrode and scale, and the sewer system to produce H_2_S. Naturally, there are already many techniques for reducing them. However, it is noteworthy that articles on the simultaneous removal of heavy metal cations and their counter-ions have been published throughout the past 10 years.

The adsorption of copper(ii) and sulphate ions onto quaternary cellulose (obtained *via* GTMAC) was studied by Gao *et al.* Sulphate anions are adsorbed through electrostatic interactions and ion exchange, while copper(ii) ions are adsorbed *via* chelation and ion exchange, involving functional groups present in quaternary cellulose. Up to pH = 5, the adsorption capabilities for copper(ii) ions and sulphate ions increase; beyond that, they decrease. Protonated H^+^ ions compete with copper ions for the active sites in quaternary cellulose at low pH levels, resulting in a reduction in copper adsorption capability. On the other hand, the H^+^ concentration decreases, and copper(ii) ion adsorption intensifies as the pH rises to 5. The hydroxide precipitation process causes the drop in copper ion adsorption above pH = 5. In the case of sulphate ions, it must be remembered that, depending on the concentration of the solution and the pH, there are three different forms. Strongly acidic circumstances cause the sulphate ions to easily protonate into sulphuric acid (VI), which reduces the electrostatic attraction to the active groups (hydroxyl and ammonium groups) and, consequently, the total amount of adsorption. Adsorption rises as a result of bisulphate ions predominating in solutions with higher pH levels because they can swap a chloride ion from quaternary ammonium salts. Adsorption diminishes above pH = 6 because part of the bisulphate ions shift to sulphate ions, which requires the exchange of two chloride ions. According to the thermodynamic analysis by Gao *et al.*, the adsorption of copper ions is exothermic and spontaneous, resulting in reduced adsorption randomness and a more ordered cellulose surface. The entropy analysis indicates that although the adsorption process of sulphate ions is exothermic, there was an increase in randomness on the adsorbent surface as it went along. The values of Δ*G* were negative, but they decreased as the temperature rose, indicating that the energy input favours the adsorption process of sulphate ions. This might be because these ions have greater difficulty exchanging with Cl-ions due to their size. Higher temperatures encourage the adsorption of sulphate ions, whereas lower temperatures favour the adsorption of copper ions. According to the pseudo-second-order kinetic model, the adsorption process of copper(ii) ions and sulphate ions reached equilibrium after 3 hours, with a notable increase in adsorption capacity during the first 45 minutes. The Langmuir isotherm (monolayer process) for copper adsorption and the Freundlich isotherm (multilayer process) for sulphate adsorption were found to have the best fits, respectively. On the other hand, the Temkin isotherm suggests that the adsorption process is more physically based. One potential drawback might be a sharp decline in adsorption capability over subsequent cycles. If copper and sulphate need to be removed together, quaternary cellulose could work well as an adsorbent. Using suitable desorption solutions, like acids or salt solutions, can help cellulose-based biosorbents regenerate, particularly after copper(ii) ions and sulphate anions have been adsorbed. Adsorption is facilitated by the structure and porosity of quaternary cellulose. Ion exchange, complexation, and electrostatic adsorbent–ion interactions all favoured adsorption.^90^

Quaternized cellulose demonstrates high potential in the adsorption of both heavy metal oxyanions and noble metal complexes. The main causes of such performance are ion exchange and electrostatic interactions with quaternary ammonium groups. Again, for all heavy metal removal processes, adsorption is pH-dependent and sensitive to competing ions. Quaternized cellulose-based adsorbent structures allow rapid kinetics, high porosity, and effective regeneration. Moreover, its ability to simultaneously remove metal cations and counter-anions, such as Cu^2+^ and SO_4_^2−^, makes quaternized cellulose a multifunctional adsorbent suitable for complex post-industrial wastewater streams.

### Removal of microplastics

Materials investigated for the adsorption of microplastics include mainly carbon-based adsorbents, especially activated carbon, biochar or graphene oxide, but also a variety of inorganic materials, clays, or metal–organic frameworks. Adsorption efficiency usually depends on the physicochemical properties of both the adsorbent and the plastic particles (surface area, porosity, surface functionality, charge, hydrophobicity, polymer type, particle size, and degree of surface ageing), but the process is also influenced by adsorption conditions, mainly pH, ionic strength, temperature, adsorbent dosage, contact time, and the presence of other contaminants.^103^ Recently, an article about adsorption of microplastics with natural polymer-based adsorbents described the use of compounds like chitosan, cellulose, alginates, pectins and their composites and their relevance in the area of microplastic removal from water sources.^104^ To date, not only various purification techniques have been proposed for the removal of microplastics, including adsorption, filtration, and flocculation, but also the mechanism is not clearly described.^104–108^ Microplastic removal is not governed by a single adsorption pathway, instead, point to a multimodal capture mechanism in which electrostatic attraction, hydrogen bonding, hydrophobic and van der Waals interactions, along with physical entrapment within a porous network, act together.^109^ Cellulose- and biopolymer-based sorbents in general, including the ones in smaller sizes, remove microplastics through the combined effect of surface chemistry and structure, especially when the adsorbent offers high surface area, abundant functional groups, and an interconnected porous framework. In practice, more than one mechanism may operate simultaneously, particularly in hydrogel and aerogel systems, where particle transport through the porous structure may be followed by mechanical retention or interfacial binding. Therefore, not all microplastic removal processes involving cellulose should be described simply as adsorption. In our work, we decided to show the potential microplastic removal by pure physical retention, flocculation and surface adsorption.

In physically driven removal systems, the porous structure of cellulose acts as a medium through which contaminated water is transported, while microplastic particles are retained within the network. According to Leppanen *et al.*, the hygroscopic nature of nanocellulose enables two water transport characteristics that, together with water flux, bring microplastics into the cellulose nanofibre hydrogel. The high specific surface area of nanocellulose then allows for the entrapment of microplastics.^108,110^ Importantly, in case of cellulose and cellulose composites, the hierarchical pore structure, the availability of hydroxyl groups and the possibility of surface functionalization seem to be the biggest advantage.^104^ Cellulose-based materials may also remove microplastics through flocculation. Faria *et al.* used bacterial cellulose-based hydrogels to efficiently entrap spherical polystyrene particles of 10 µm in diameter, achieving 93–96% efficiency.^108^ The bacterial cellulose was used as a flocculating agent and exhibited enhanced flocculation activity of PS particles with a mean diameter of 100 µm.^108^

Nevertheless, the unmodified cellulose does not exhibit any affinity for microplastic particles, therefore, to enhance the performance of pure cellulose, quaternization has been used in a few recent articles on microplastic removal. Quaternized cellulose aerogels were used by Zhuang *et al.* for the removal of small poly(vinyl alcohol) particles with an efficiency of 146.38 mg g^−1^.^7^ The adsorption behaviour of the prepared aerogels was described by the Langmuir isotherm and pseudo-first-order and pseudo-second-order adsorption kinetics models. The negatively charged microplastics in that case enabled electrostatic interactions with the cationic adsorbent, resulting in an almost 10-fold increase in adsorption compared with non-functionalized CNF aerogels. In other studies, EPTMAC was again used to modify cellulose derived from rice straw, however it was also combined with chitosan to create a quaternary ammonium salt-modified cellulose/chitosan composite aerogel.^111^ The adsorption efficiency of polystyrene microplastics with a mean diameter of 1 µm exceeded 80% and was evaluated across pH values (4–10) and temperatures (20–40 °C). High adsorption efficiency is maintained during subsequent regeneration cycles (with only a slight decrease after multiple cycles), indicating that the material is stable and suitable for repeated use. Moreover, the authors showed that PS particles can also be easily removed under real conditions, including removal from river, lake, and tap water. The adsorption process fits pseudo-second-order kinetics, with a theoretical maximum adsorption capacity of 729.9 mg g^−1^ estimated from the Langmuir isotherm.

The functional groups present on the surface of nanocellulose also significantly influence adsorption. Unmodified cellulose contains hydroxyl groups, which can form hydrogen bonds with the surface of microplastics. Furthermore, van der Waals forces and hydrophobic interactions occur between the adsorbent surface and the microplastic particles. Porosity and a large number of adsorption sites increase the likelihood of microplastics coming into contact with the adsorbent surface, which translates into greater efficiency in removing contaminants.^109^ It is worth emphasizing the importance of quaternization of nanocellulose. In the case of quaternary cellulose aerogels, microplastic removal was primarily driven by electrostatic interactions between the microplastics and the adsorbent. This modification increased the adsorption capacity tenfold compared to unmodified aerogels.^7^

The strength of any of the interactions that govern the microplastic removal can be increased by incorporating another components into the cellulose matrix. One of the examples described in recent literature is chitosan. The arrangement of functional groups and the morphology of the adsorbent interact to regulate the availability of active sites, thereby influencing contaminant capture and, in turn, adsorption efficiency. One of the most important structural features of the adsorbent is its extensive network of fibres and pores. The hygroscopicity of nanocellulose means that water contaminated with microplastics can be effectively transported through the hydrogel or aerogel matrix, facilitating their transport into the adsorbent interior. Furthermore, the large specific surface area enables the physical retention of microplastic particles within the nanocellulose network.^110^

The performance of quaternary cellulose adsorbents arises from the combination of high surface area, tuneable surface chemistry, and porous architecture, which together promote microplastic capture through physical entrapment, and interactions such as hydrogen bonding, electrostatic attraction, and other surface-dependent forces. The reviewed studies indicate that microplastic removal by quaternary cellulose adsorbents cannot be assigned to one universal mechanism. Larger particles may be removed mainly through filtration, retention, or flocculation, whereas smaller and surface-charged particles may be more strongly influenced by interfacial adsorption. In quaternized materials, electrostatic attraction is an important contribution, but hydrogen bonding, van der Waals interactions, hydrophobic interactions, and physical confinement may also participate. In quaternized cellulose materials, permanent cationic groups enhance affinity toward negatively charged or oxidized microplastic surfaces, while the porous framework facilitates particle transport and retention. Overall, the available evidence indicates that microplastic removal by cellulose-based materials is structure-dependent and results from the synergy between tailored surface functionality and porous architecture rather than from a single universal adsorption mechanism.

### Adsorption of gaseous carbon dioxide

It is important to note that carbon dioxide adsorption is another application for quaternary cellulose. The issue of CO_2_ emissions is worsening due to rising energy use, which intensifies the greenhouse effect. Therefore, it is essential to determine the most biocompatible method of CO_2_ capture. Many substances have been employed for this purpose over time, including zeolites, activated carbons, and ethanolamine.

However, a cellulose-based substance created by Hou *et al.* seems to be by far the most effective in absorbing CO_2_. To functionalize the cellulose, they employed CHPTAC. Adsorption of carbon dioxide follows pseudo-second-order kinetics and the Langmuir isotherm, indicating a monolayer process. When CO_2_ is present at 400 ppm, the adsorbent is 90% saturated. The adsorption process was not favoured by extremely low or extremely high relative humidity; hence, the best results were achieved at 66% humidity. The interfacial hydrophilicity most likely significantly affects the outcomes of the adsorption process. The aforementioned research could serve as a basis for developing novel SILs that absorb CO_2_ more effectively.^88^ CO_2_ capture on quaternary cellulose describes the mechanism of moisture swing, combining a fibrous structure, a high specific surface area, and the presence of cationic groups. The number of available active sites depends on the density and distribution of ammonium groups, while gas transport and contact are improved by the adsorbent's porous morphology. Relative humidity has a crucial impact on CO_2_ adsorption, regulating both site availability and competitive water adsorption. In summary, the effectiveness of direct CO_2_ capture from the air is determined by the interaction between the structure of the cellulose material and its modification. Moisture facilitates adsorption by promoting the production of carbonates and bicarbonates. Adsorption follows pseudo-second-order kinetics and the Langmuir model, explaining the initial rapid uptake that slows as active sites become occupied. The availability of these sites and effective mass transport are enabled by the adsorbent's porous structure.

The strong potential of quaternary cellulose for CO_2_ capture addresses the need for sustainable greenhouse gas removal strategies. Such structures exhibit rapid, monolayer CO_2_ adsorption, achieving high efficiency even at low CO_2_ levels. Adsorption efficiency is strongly influenced by the humidity of the air being purified, with optimal conditions at intermediate humidity.

### Adsorption mechanisms

The adsorption mechanisms reported for different pollutants indicate that quaternized cellulose does not work in a single and universal interaction pathway. In most of the described systems the main driving force for adsorption is the electrostatic attraction between negatively charged adsorbates and the permanently cationic ammonium or phosphonium groups introduced on the cellulose surface. At the same time, the reported studies show that this dominant mechanism is often accompanied by additional contributions from ion exchange, hydrogen bonding, van der Waals interactions and, in selected cases, hydrophobic effects. Hence, the adsorption behaviour of quaternized cellulose has to be considered as the consequence of several coexisting interactions, the relative importance of which is depending on the structure of the adsorbate and the physicochemical conditions of the system.

The proposed mechanisms are largely consistent in showing the central role of electrostatic attraction and ion exchange for dyes, surfactants, disinfection by-products and several inorganic anions. However, the reviewed studies also clearly show that the efficiency of adsorption is highly affected by pH, ionic strength, pollutant speciation, and competition with other ions present in the solution. In the adsorption of dyes, the differences in the affinity of structurally related compounds are due to additional interactions such as hydrogen bonding and, in some cases, to more specific intermolecular effects. In surfactant systems, the adsorption process is further stabilized by the interactions involving the hydrophobic tail. The final performance of the adsorbing of chlorinated acids, chromate species, sulphate or Au(CN)_2_ depends not only on the presence of cationic sites, but also on the easiness of ion exchange and the accessibility of those sites under given solution conditions. These observations indicate that quaternized cellulose is particularly well-suited for removal of anionic pollutants, although the surface charge alone cannot predict the degree of adsorption.

When it comes to proteins, carbon dioxide and microplastics, the results indicate that the importance of morphology and structural accessibility is similar to surface chemistry. Electrostatic attraction is the dominant mechanism for protein adsorption, but it is also affected by the protein charge state, pH relative to the isoelectric point, and the nanofibrillar or porous structure of the adsorbent which controls diffusion and access to active sites. For CO_2_, the mechanism is different from that in aqueous systems, and is related to moisture-assisted adsorption, where ammonium functionality, hydrophilicity of the interface, and porous morphology jointly determine gas uptake. The case of microplastic removal seems the most straightforward one for multimodality as it involves not just electrostatic attraction but also hydrogen bonding, hydrophobic and van der Waals interactions, and physical entrapment within the porous network. The mechanisms presented in the reviewed studies together suggest that the adsorption performance of quaternized cellulose is modulated not only by cationization but also by the combined effect of surface functionality, material architecture, and pollutant-specific interactions.

## Challenges and limitations

Even though it has great potential for removing a variety of organic and inorganic compounds, including those of emerging concern, the large-scale practical use of quaternary cellulose in adsorption is still limited by several issues. Its implementation may be feasible if key concerns such as stability, reusability, scalability, and environmental impact are addressed.

A crucial factor influencing the long-term efficacy of quaternary cellulose, especially in pollutant adsorption, is its stability. The impact of the degree of substitution of quaternary cellulose on mechanical strength was evaluated using tensile and burst strength tests. The results clearly indicated increases of 6.17% and 11.68%, respectively, at a degree of substitution of 0.51.^50^ The stability of adsorbents depends not only on the structural groups available in cellulose but mainly on its structure. One way to preserve the structural integrity and prevent collapse or compaction of the delicate three-dimensional structure of quaternary cellulose is through efficient cross-linking, because quaternary groups themselves don't provide additional stability to the system. Since cross-linking always affects the adsorption process, any changes in chemical structure need to be rationally designed.^76^

In hydrogel systems obtained by click reactions, chemical stability is significantly higher than in classical systems. An interpenetrating polymer network (IPN) provides stronger chemical bonds that are resistant to dissociation, although environmental factors such as ionic strength and competing ions in real wastewater can still pose a challenge to long-term stability. Covalent networks increase stability compared to conventional ones, which can undergo swelling–deswelling fatigue during cyclic use, leading to pore expansion or collapse, which reduces the available active sites for pollutants.^61^ Research is currently underway to optimize these factors to obtain quaternary cellulose with balanced chemical and mechanical stability.

The reusability of quaternary cellulose is essential, especially considering its economic value, but also from an environmental point of view. As far as biodegradability is concerned, cellulose is favoured; its possible degradation during the removal process is a disadvantage. The material should be able to last for several processing cycles in order to be useful. Not much research demonstrates regeneration and reusability potential, even though only a few cycles are usually considered. According to the literature, they can be regenerated and reused for up to six cycles without loss of capacity if produced correctly.^86^ Depending on the type of pollutant, several regeneration methods are available; the desorption conditions must be chosen carefully. For removing trapped heavy metals or anions from the surface of quaternary cellulose, weak acid solutions (diluted sulphuric acid (VI)) or chelating agents (EDTA) can restore active sites with minimal damage to the adsorbent.^87^ Although QACC efficiency decreases with the number of cycles, reuse efficiency is usually very high in the first few cycles, as shown in Table 6. Interestingly, not every study performed on adsorption provides data on the possibility of reuse.

**Table 6:** Recovery parameters of quaternary cellulose after adsorption

Name of adsorbent	Regeneration solution	Cycles of adsorption	Removal efficiency (%)	Ref.
CCF2	11 M HCl	8	Approximately 70	63
QACC	0.1 M NaOH and 0.1 M H_2_SO_4_	6	96	86
QACC	1 M HCl	3	99	87
CNFs aerogel	0.1 M NaOH	4	84.9	16
QACC	1 M NaOH	3	99	82

It is worth noting that the economic value of adsorbents primarily depends on their reusability. Research performed so far is quite promising. During repeated use, not only may cross-linking agents or reinforcing chemicals leak out, endangering the adsorbent's integrity and possibly causing secondary pollution, but an ionic regeneration solution may also influence the adsorbent's chemical structure. Therefore, more effort should be put not only into the regeneration process itself but also into confirming that the regeneration solution doesn't change the composition (*e.g.*, a strong base can act as an ion-exchange resin for chloride anions).

The available research is conducted in controlled laboratory settings, which do not accurately reflect process complexity (ionic strength, competing pollutants, *etc.*). Moreover, it is difficult to compare existing solutions because each material is produced differently and can exhibit varied sizes and shapes. Definitely, the long-term and economical use of the QACC depends on the evolution of facile regeneration techniques.

For the widespread use of quaternary cellulose adsorbents, the adsorption process must be economical and scalable. Cellulose is abundant and can usually be obtained from agricultural waste; therefore, its advantages over other polymers are widely acknowledged. Scientific research benefits from low purchasing costs and easy access; however, the functionalization process incurs costs. Hence, it is necessary not only to obtain the desired efficiency but also to evaluate the functionalization scalability. Production costs increase with the use of quaternary agents (mainly CHPTAC) or cross-linking agents. It is important to balance the materials' adsorption properties with economic considerations; however, the utilization of cellulose waste significantly influences economic factors.^112^

According to the reviewed studies, quaternary cellulose, as an adsorbent, offers several environmental benefits compared with commercial or more traditional adsorbents (inorganic materials or synthetic polymers). Utilizing cellulose, especially that derived from renewable biomass or agricultural waste, most of all reduces dependence on non-renewables.^80^ Due to their non-toxicity and biodegradability, there is a reduced risk of secondary contamination at the adsorbent's end of life.

In general, the removal of organic contaminants by quaternary cellulose adsorbents can be achieved under mild conditions, without hazardous chemicals or labour-intensive processes. Even after several adsorption and desorption cycles, quaternary cellulose exhibits very low leaching, unlike metallic adsorbents, which can release harmful ions. The high adsorption efficiency, low toxicity, and biodegradability of these materials make them attractive options in treatment technologies, especially for removing hazardous anionic compounds from contaminated natural waters and industrial wastewater. However, to fully measure the environmental benefits, including energy input during synthesis, chemical consumption for functionalization, and waste management, life-cycle assessment and large-scale analysis are necessary.

## Strengths – weaknesses – opportunities – threats (SWOT) of the quaternary cellulose adsorbents

The development of quaternary cellulose-based adsorbents presents a broader shift toward functional, bio-derived materials with potential in the removal of a variety of contaminants. Nevertheless, the newly developed field remains simplified, laboratory-scale, and idealized; that's why we decided to present a strategic evaluation of its strengths, weaknesses, opportunities, and potential threats (SWOT analysis, presented in Fig. 10).

**Fig. 10:**
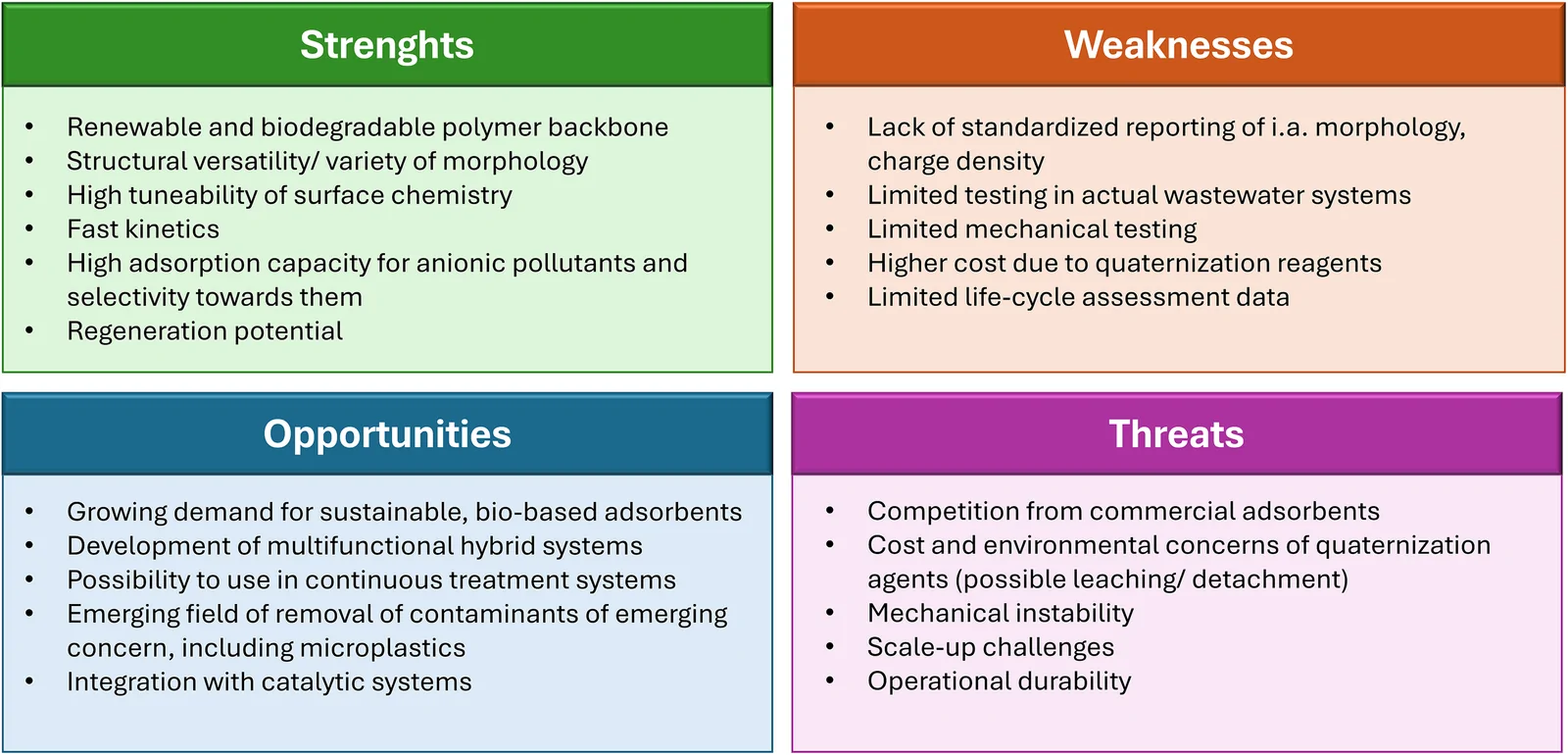
SWOT analysis for the use of quaternary cellulose adsorbents.

The main strength of quaternary cellulose adsorbents is their renewable, biodegradable backbone, which offers a significant advantage over synthetic polymeric adsorbents. Modification with quaternary ammonium (or phosphonium) groups enables a change in the surface charge, allowing additional removal mechanisms to take place, *e.g.*, electrostatic interactions, especially important in the case of the removal of a wide spectrum of negatively charged pollutants, including anionic dyes, surfactants, chlorinated acids, proteins, and even microplastics. Given that a variety of structures can be obtained, ranging from aerogels and hydrogels to microspheres and nanofibres, it is possible to design appropriate, target-specific adsorbents. Even though most scientific descriptions show a similar quaternary structure, the versatility of surface modification, including varied alkyl chain lengths, branched chains, additional unsaturated bonds, or even anion exchange (cellulose-supported ionic liquids), offers great promise for developing an interesting platform for contaminant removal, enabling good selectivity and adsorption efficiency. Like any adsorbent, quaternary cellulose has its limitations. The main problem is the large-scale implementation of those systems, which is largely due to a lack of standardized reporting of their key properties: morphological (size, shape, porosity), structural (charge density, degree of crosslinking), and the repeatability of the quaternization reaction. The cost of the quaternization reaction is also high, not only economically but also in terms of the environmental burden it can pose. Importantly, almost all removal studies are conducted under strictly simplified laboratory conditions that do not capture the complexity of real wastewater, and, as is well known, this can drastically alter removal efficiency and regeneration characteristics. Another point is the limited knowledge about the mechanical properties of quaternary cellulose systems.

At the same time, significant prospects are present for the advancement of the quaternary cellulose adsorbents' field, especially when considering the increasing demand for sustainable and biodegradable alternatives to synthetic sorbents and flocculants, especially when cellulose wastes from agriculture or industry are considered. Emerging problems, such as microplastic removal and the selective recovery of precious metal complexes, indicate promising application areas. Furthermore, the great promise lies in the development of hybrid or cross-linked systems that combine the advantages of quaternary cellulose with those of various functional or catalytic components, or that improve their mechanical properties. Given the external threats that may hinder further development in the field of quaternary cellulose adsorbents, the competitiveness of commercial adsorbents is especially crucial. Commercial adsorbents, carbon, biopolymer, or synthetic-based ones are well known, well-defined, demonstrate appropriate mechanical properties, and possess favourable properties when it comes to their entire adsorption-cycle properties. For now, research on quaternary adsorbents focuses excessively on adsorption capacity, with little consideration of efficiency, regeneration, mechanical stability, durability, and life-cycle analysis.

Taken together, the SWOT analysis indicates that quaternary cellulose adsorbents have strong conceptual and environmental foundations; however, systematic efforts to overcome structural, economic, and operational barriers are still urgently needed. The eventual success of this material will depend not only on enhancing adsorption performance but also on integrating durability, scalability, and sustainability considerations into the design process.

## Design guidelines for future quaternary ammonium adsorbents

Quaternary-functionalized cellulose adsorbents are a great example of how targeted chemical modification can transform a widely distributed biopolymer into a highly versatile platform for removing a range of pollutants. The final performance against targeted pollutants is generally governed by a delicate balance among structural properties such as morphology, degree of substitution with quaternary ammonium (or phosphonium) groups, and mechanical properties. The materials must, moreover, be tailored to the size and transport characteristics of contaminants. In general, hierarchically porous aerogels and swollen hydrogels offer advantages such as rapid uptake and high capacity, while microspheres or reinforced networks provide easier handling and greater regeneration stability. Importantly, the performance of quaternary cellulose adsorbents in laboratory research on single-pollutant removal cannot be the sole proof of their practical applicability; a careful evaluation in multi-ion systems and in real wastewater is essential to assess their potential for final application. While challenges remain in durability, functionalization cost, and scalability, the interesting tuneability of quaternary cellulose offers a strong foundation for rational, application-specific design, positioning these materials as promising candidates for sustainable remediation technologies. To facilitate the transition of quaternary cellulose adsorbents from laboratory research to real-world environmental technology, we prepared a design framework for future research that integrates not only structural parameters and their performance, linked to the targeted pollutant and various removal mechanisms, but also performance validation under real-world conditions. The framework presented in Fig. 11 integrates the design steps to guide the future development of sustainable quaternary cellulose adsorbents for practical use.

**Fig. 11:**
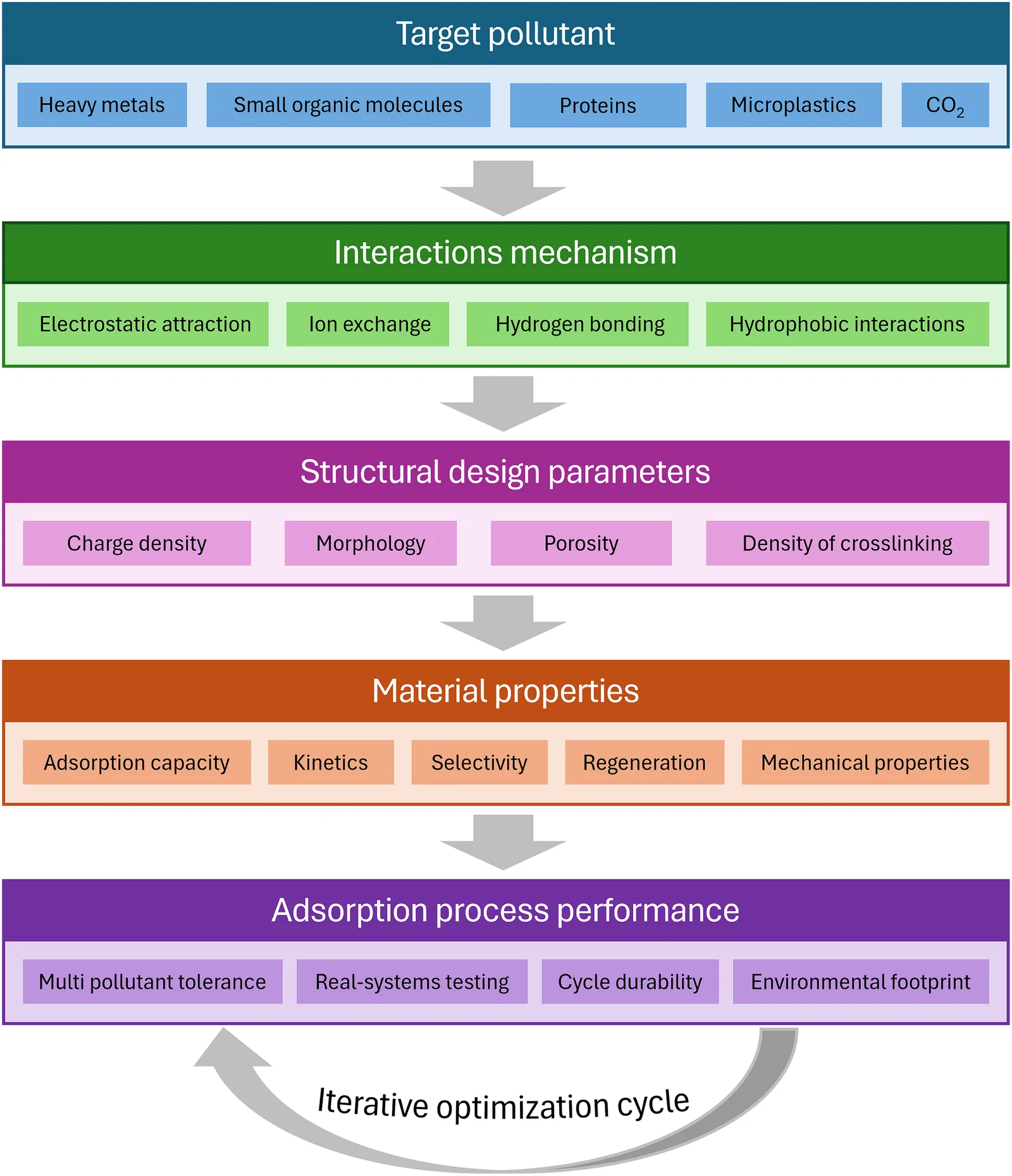
Design frameworks for quaternary cellulose adsorbents.

The first step is the identification of the contaminants. Different pollutants: heavy metal ions, small organic molecules like dyes, proteins, or microplastics, require varied interactions and structural features. The dominant adsorption mechanisms, typically electrostatic interactions, ion exchange, hydrogen bonding, or hydrophobic interactions, guide the selection of specific functional groups, the degree of functionalization or morphology, and therefore the entire synthetic route. In this sense, the structural and physicochemical design of the adsorbent must be thought through, including the density of cationic groups, morphology, size and shape (fibrous, spherical, porous, or hydrogel structures), porosity, and the control over the accessible active sites, which might be influenced by the crosslinking density. From a materials point of view, future studies should focus on establishing quantitative correlations between the degree of substitution and accessible charge density, morphology, and eventual crosslinking density, as well as key performance characteristics such as adsorption capacity, kinetics, and selectivity, especially in multi-component systems, regeneration efficiency, and mechanical durability. Standardized reporting of the degree of substitution and the accessible charge density seems essential to enable meaningful comparisons and to ease optimization across different classes of pollutants. Finally, the adsorption performance needs to be validated under realistic environmental conditions and with a thorough, systematic evaluation of the regeneration process and stability. This should involve studies that extend beyond 10 adsorption–desorption cycles, structural characterization after repeated use, analysis of potential loss of functional groups, and a full assessment of regeneration and processing. Simultaneously, ongoing research on the preparation of quaternary cellulose adsorbents should continue, particularly regarding greener quaternization routes and process scale-up to achieve economic and environmental sustainability. Overall, such an iterative design approach seems crucial to ensure that the performance, durability, and environmental footprint of quaternary cellulose adsorbents can be realistically optimized for real-world remediation applications.

## Conclusions

The development and application of quaternary cellulose structures and nanostructures represent a step forward in sustainable environmental water treatment. Quaternary cellulose demonstrates high efficiency in adsorbing a variety of pollutants, such as dyes, antibiotics, heavy metals, and even microplastics. The field remains predominantly at a laboratory scale. Most studies are conducted under simplified, controlled conditions using model pollutant solutions that don't represent real wastewater. There is a clear lack of scale-up investigations. An increasing amount of research is being published each year about the adsorption of pollutants in water by quaternary cellulose. Quaternary cellulose remains underutilized, despite encouraging results. Quaternary cellulose demonstrates superior adsorption capacity, reusability, and alignment with green chemistry principles; however, there is currently no reliable method to predict its future fate. The adsorption of organic anionic molecules generally exceeds that of inorganic substances. This may be affected by increased chemical affinity between the adsorbent and the organic adsorbate. The literature review indicates that the adsorption of chemicals using quaternary cellulose is an emerging field, with numerous opportunities for further exploration.

The performance of quaternary cellulose in real wastewater systems is still insufficiently understood (pH, ionic strength, competing contaminants). The cost and environmental impact of the quaternization reagents used are crucial for this topic. Further in-depth research is needed to determine the cost-effectiveness and sustainability of modification methods that could be widely applied. Research on repeated regeneration cycles, structural degradation, and comprehensive durability studies is still limited. For materials intended for sustainable water treatment, operational lifetime, durability, and long-term evaluations are crucial for practical environmental applications.

Future research may also focus on developing a multifunctional quaternary cellulose system that incorporates other biopolymers, inorganic nanoparticles, or responsive materials. Such advancements will surely enable the development of materials that dynamically respond to environmental stimuli and contaminants. Further progress in research will involve a strategic shift in research priorities. Instead of focusing on increasing adsorption capacity under ideal conditions, researchers should concentrate their efforts on scalability, regeneration process efficiency, and testing actual wastewater samples. The continuous development of hybrid and multifunctional materials may offer improved performance. However, increased structural complexity must be critically evaluated in terms of cost, recyclability, and environmental impact.

Quaternary cellulose as an adsorbent combines renewability and strong electrostatic affinity for many organic compounds, mainly anionic ones. However, its practical application remains limited due to the limited number of studies on long-term durability, insufficient validation in complex wastewater samples, and unresolved issues related to cost-effectiveness and sustainable life cycle compared to commercial adsorbents. In summary, quaternary cellulose offers great promise for future water purification technology. With interdisciplinary research and industry collaboration, it has the potential to become a popular commercial adsorbent within the next decade or so.

## Author contributions

Bartosz Fabiszczak: conceptualization, methodology, data curation, formal analysis, writing – original draft, writing – review and editing, visualization. Roksana Markiewicz: conceptualization, methodology, data curation, formal analysis, writing – original draft, writing – review and editing, project administration, funding acquisition.

## Conflicts of interest

There are no conflicts to declare.

## Data Availability

No primary research results, software or code have been included and no new data were generated or analysed as part of this review.
